# Efficient assays for measuring inhibition of penicillin binding protein 3 (PBP3) transpeptidases

**DOI:** 10.1039/d6cb00304d

**Published:** 2026-11-10

**Authors:** Helen G. Smith, Ignacio E. Tobal, Stephen A. Marshall, Alistair J. M. Farley, Christopher J. Schofield

**Affiliations:** a Department of Chemistry, University of Oxford 12 Mansfield Road Oxford OX1 3TA UK helen.smith@chem.ox.ac.uk christopher.schofield@chem.ox.ac.uk; b Ineos Oxford Institute for Antimicrobial Research, University of Oxford 12 Mansfield Road Oxford OX1 3TA UK

## Abstract

β-Lactam antibiotics, which are amongst the most highly used anti-infective medicines, function *via* inactivating covalent reaction with the nucleophilic serine residue of transpeptidases (penicillin binding proteins, PBPs). Few methods for monitoring the rate of PBP acylation by β-lactams are reported, limiting the development of new inhibitors. We describe a fluorescence-based method employing release of umbelliferone from a cephem (FC5) for the efficient determination of the second order rate constant (*k*_inact_*/K*_I_) for PBP inhibition. The method is shown to be suitable for efficient generation of inhibition rate constants for multiple PBP3s. The FC5 probe used is also suited for β-lactamase assays, allowing multiple enzyme (sub)classes to be screened using a single reporter compound. The results of mass spectrometry-based assays measuring the efficiency of covalent reaction with PBP3s compared to meropenem support the results obtained from the fluorescence-based assays. X-ray crystallographic analyses of *P. aeruginosa* PBP3 following reaction with ertapenem, doripenem and biapenem inform on interactions leading to difference in the rates of their reactions with PBP3. The PBP3 methods will be useful in efforts to develop both novel β-lactam and non-β-lactam transpeptidase inhibitors with improved activity and β-lactamase resistance properties.

## Abstract 2

A fluorescence-based method employing the release of umbelliferone from a cephem allows efficient determination of the second order rate constant (*k*_inact_/*K*_I_) for inhibition of the transpeptidase penicillin binding protein PBP3.

## Graphical abstract

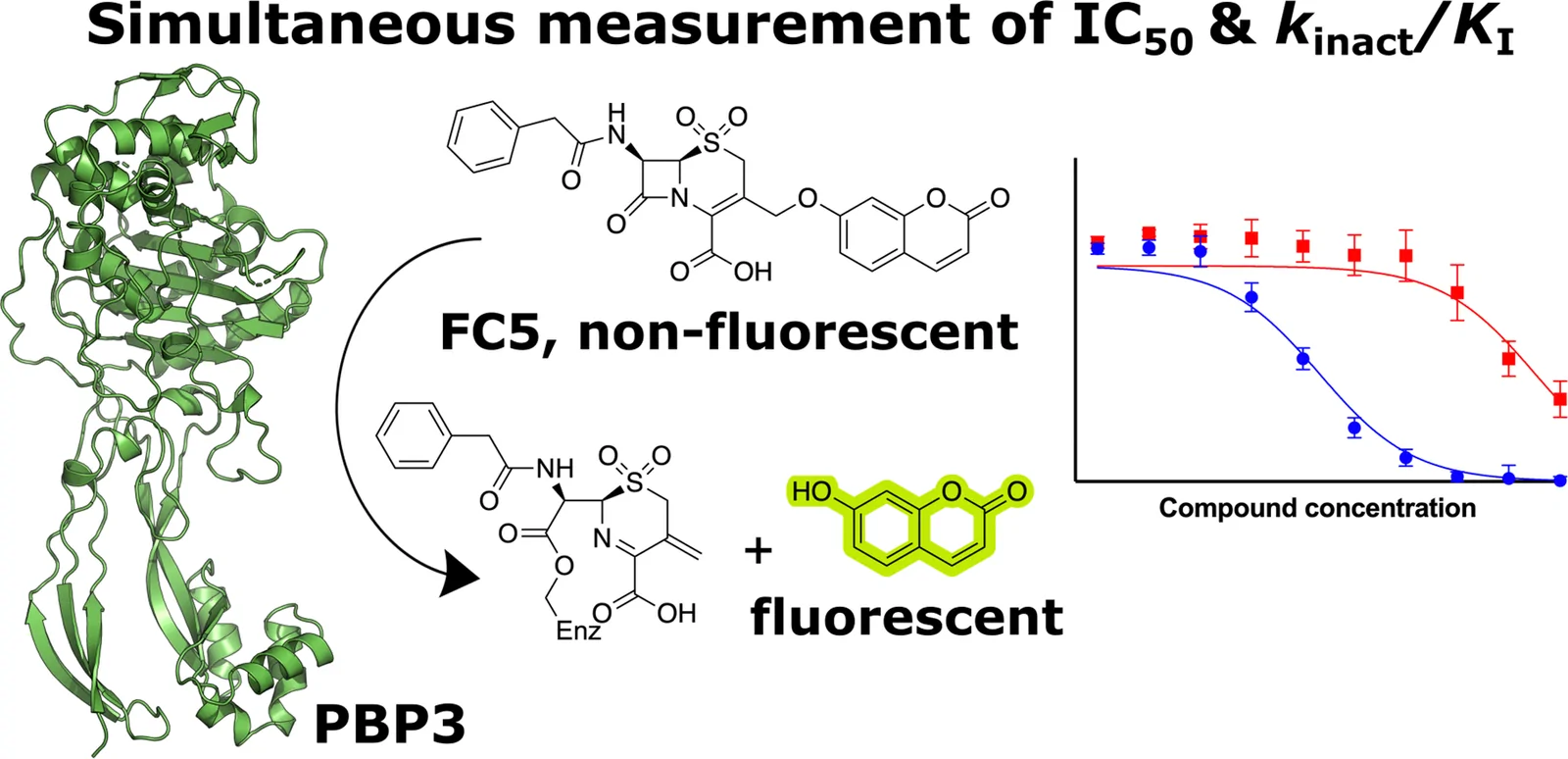

## Introduction

Despite being discovered almost a century ago, β-lactams remain some of the most important antibiotics,^1^ including for treatment of the ESKAPE panel of Gram-negative bacteria for which novel therapies are urgently required.^2,3^ β-Lactam antibiotics inhibit transpeptidase penicillin binding proteins (PBPs) *via* covalent modification of their nucleophilic active site serine residue.^4^ PBPs in Gram-negative bacteria, play an essential role in the formation and maintenance of the peptidoglycan (PG) layer. PG is comprised of strands of repeating *N*-acetylglucosamine-*N*-acetylmuramic acid (GlcNAc-MurNAc) disaccharide subunits, each MurNAc sugar is functionalised with a pentapeptide stem (usually l-Ala-d-Glu-*m*-DAP-d-Ala-d-Ala in Gram-negative bacteria).^5^ Transpeptidase PBPs catalyse the formation of ‘4-3-crosslinks’ between the d-Ala and *m*-DAP residues of adjacent pentapeptide stems. These crosslinks are essential for formation of the mature PG mesh structure, which maintains the structural integrity of the bacterial cell.^5^

Many Gram-negative bacteria have four high molecular weight PBPs with transpeptidase activity: PBP1a and PBP1b are dual functional transpeptidases and transglycosylases; PBP2 and PBP3 are monofunctional transpeptidases.^5^ Of these, PBP3 is often identified as a key target enzyme when screening for novel transpeptidase inhibitors – PBP3 plays a key role in cell division and its inhibition can lead to apoptosis.^6,7^

Pioneering SAR-studies on the development of PBP inhibiting antibiotics were principally driven by microbiological assays. Despite PBPs being important antibacterial targets, few efficient assays have been developed to measure PBP inhibition in isolated form. This is likely due to the lack of recombinant methods for protein production in the pioneering times and, more recently, challenges in isolating or synthesising the natural PBP substrates. PBP catalysed crosslinking of natural peptidoglycan substrates has been investigated employing a coupled assay system wherein the alanine released on reaction is converted to a fluorescent signal through the use of a d-amino acid oxidase and horseradish peroxidase.^8,9^ A widely employed method for measuring PBP inhibition uses Bocillin™ FL, a commercially available BODIPY-functionalised penicillin derivative.^10,11^ Bocillin™ FL can be used to measure either pIC_50_ values or, in an alternative format, to measure *k*_inact_*/K*_I_ values.^11^ As β-lactams bind covalently and (near) irreversibly to PBPs, their inhibition is time dependent. Measurement of *k*_inact_*/K*_I_, a second-order rate constant, provides information on both reactivity (*k*_inact_) and binding affinity (*K*_I_) and is an important parameter in understanding the activity of covalently reacting inhibitors.^11–13^

To enable the efficient analysis of improved PBP inhibitors, we sought to develop assays to derive second order rate constant *k*_inact_*/K*_I_ values from endpoint pIC_50_ studies.^14^ Here we describe the development and implementation of fluorescence-based PBP3 assays employing the release of umbelliferone from a cephem, previously used in β-lactamase assays,^15,16^ for efficient determination of *k*_inact_*/K*_I_ values for PBP3s. The solution assay results are supported by protein observed electrospray ionisation mass spectrometry results. Crystal structures of *P. aeruginosa* PBP3 in complex with different inhibitors inform on possible reasons for differences in inhibitor reactivity. The methods developed will be useful for studies on other PBPs.

## Results and discussion

### Assay development

Miyahisa *et al.* have described a method that enables calculation of *k*_inact_*/K*_I_ values for covalently inactivating enzyme inhibitors from endpoint IC_50_ values *via* implementation of a competitive binding assay.^17^ This method requires the use of a probe which binds irreversibly to the target enzyme and that binding is only measured once irreversible covalent reaction has occurred.^17^ As cephalosporins can be potent PBP3 inhibitors, we elected to test three cephalosporin derivatives previously utilised for the *in vitro* study of nucleophilic serine- (and metallo-) β-lactamases.^15,16^ These probes, FC3, FC4 and FC5 (SI Fig. S11), comprise a cephalosporin core functionalised at their C3 position with umbelliferone.^15,16^ The highly fluorescent umbelliferone is rendered near non-fluorescent when its phenolic oxygen is ether-derivatised as in the FC probes. Following opening of the β-lactam ring of the FC probes, release of umbelliferone occurs producing a fluorescence readout^15,16^ that, unlike fluorescence polarisation probes such as Bocillin™ FL, should only produce a signal following covalent bond formation with the PBP nucleophilic serine. We therefore considered that the FC probes could be applied to PBPs to allow development of efficient endpoint assays for PBPs, informing on the efficiency of inactivation.

Miyahisa *et al.* (SI Fig. S2) have applied a simplified kinetic model for enzyme inactivation and eqn (1), to enable calculation of *k*_inact_*/K*_I_ values from IC_50_s determined in endpoint assays.^17^1
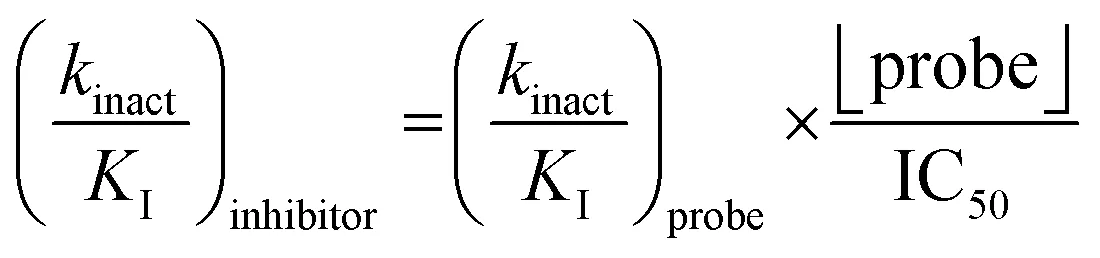


Testing of *A. baumannii* PBP3 with FC5 indicated that it reacted very efficiently with the target protein (SI Fig. S4) compared to FC3 and FC4. Consequently, FC5 was selected for further evaluation with PBP3s. Initially, the *k*_inact_*/K*_I_ of FC5 was determined for PBP3s from Gram-negative bacterial species of clinical interest (*A. baumannii, E. coli, K. pneumoniae, P. aeruginosa*) *via* measurement of the increase in fluorescence intensity on reaction with different concentrations of FC5 at a fixed PBP3 concentration over 2000 seconds (SI Fig. S4). The method used by de Munnik *et al.* in studies on the l,d-transpeptidase Ldt_Mt2_ from *Mycobacterium tuberculosis* using alternate probe compounds was used to enable calculation of (*k*_inact_*/K*_I_)_probe_, (*k*_obs_)_probe_ and *t*_1/2_ values.^14^ The *t*_1/2_ measurements enabled determination of the reaction endpoints, with >97% FC5 reaction for all the PBPs, with the exception of *P. aeruginosa* PBP3 where a slower reaction time necessitated selection of an endpoint with >95% occupancy (Fig. 1 and SI Fig. S4, S5). The *k*_obs_ (500 nM FC5) for *K. pneumoniae* and *E. coli* PBP3 are almost double the values for *P. aeruginosa* and *A. baumannii* PBP3. Consequently, a longer reaction time was employed for the *P. aeruginosa* and *A. baumannii* PBP3, because the corresponding *t*_1/2_ is higher.

**Fig. 1:**
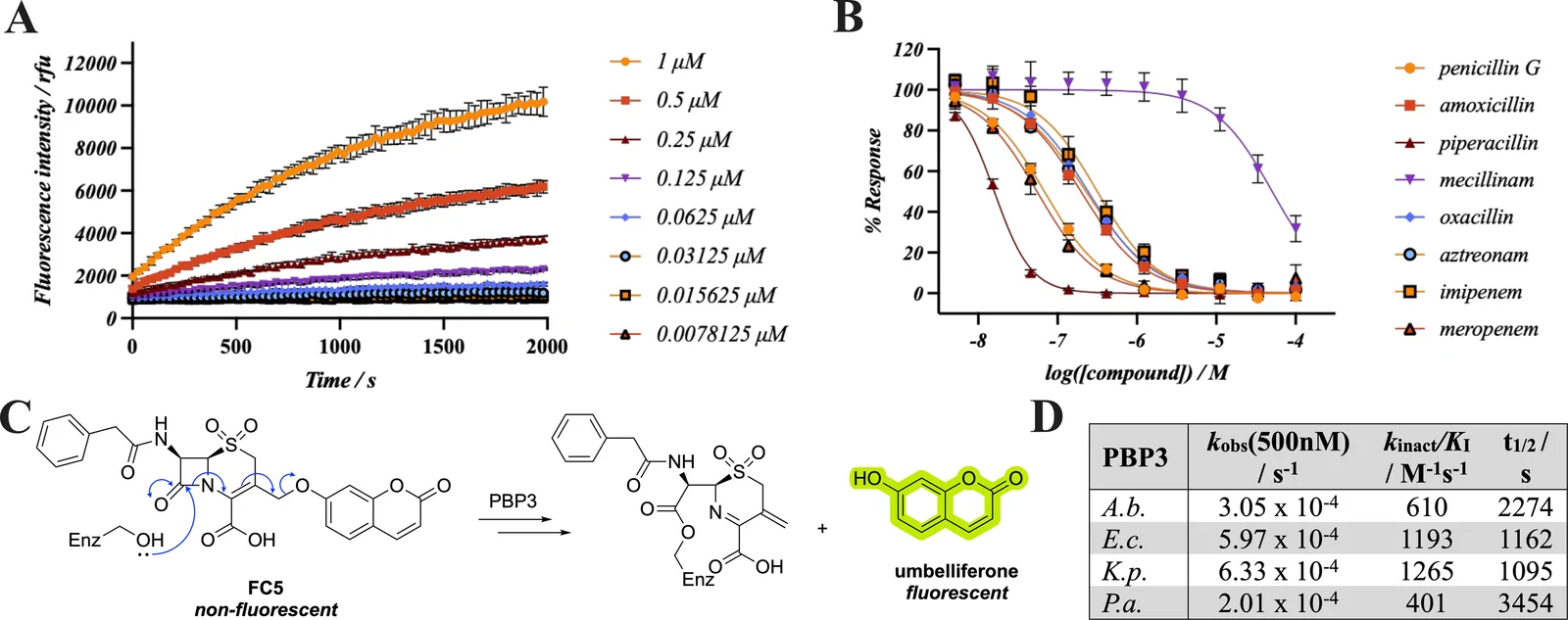
(A) Fluorescence-based assay for penicillin binding protein transpeptidase activity. Representative reaction progress curves for varied concentrations of FC5^15,16^ with *A. baumannii* PBP3 (50 nM) enable *k*_obs_ calculation; (B) representative pIC_50_ curves for β-lactams with *A. baumannii* PBP3; (C) outline mechanism for reaction of FC5 with PBP3, involving elimination of umbelliferone from the C3 position; (D) following initial optimisation, *k*_obs_, *k*_inact_*/K*_I_ and *t*_1/2_ were measured for PBP3 from *A. baumannii*, *E. coli*, *K. pneumoniae* and *P. aeruginosa* at 50 nM with FC5^15,16^ probe (500 nM) in 50 mM potassium phosphate buffer, pH 7.4.

### Inhibitor screening using the FC5 assay

Once optimised assay conditions had been determined, a set of known PBP inhibitors was screened to investigate assay robustness. Representative compounds from the penicillin, monobactam, carbapenem, and cephalosporin β-lactam antibiotic classes were tested. Mecillinam, an amidinopenicillin that is reported to be selective for PBP2, was included for comparison (Table 1 and SI Fig. S6–S9).^18,19^

**Table 1:** pIC_50_ and *k*_inact_/*K*_I_ values measured for PBP3s. PBP3s (50 nM) from *A. baumannii* (*A.b.*)*, E. coli* (*E.c.*)*, K. pneumoniae* (*K.p.*) and *P. aeruginosa* (*P.a.*) were assayed using FC5^15^ (500 nM) in 50 mM potassium phosphate, pH 7.4. Covalent reaction competition assays with meropenem employing mass spectrometry (MS) were undertaken with *A. baumannii* PBP3: the inhibitor of interest (1 mM) was co-incubated with meropenem (1 mM) and *A. baumannii* PBP3 (100 µM) for 10 minutes; the products were then analysed by UPLC-ESI-Q-TOF MS. The results are categorised as: N/A – overlapping peaks obscure quantification of results; none – only the meropenem adduct observed; high – the major peak is the adduct with the compound under investigation; low – the major peak is the meropenem derived adduct. The pIC_50_ and *k*_inact_*/K*_I_ values are reported as the mean average ± the standard deviation of three independent experiments. See Fig. S10 for compound structures

	pIC_50_				*k*_inact_/*K*_I_/M^−1^ s^−1^				MS assays: extent of competition with meropenem
	*A. b.*	*E. c.*	*K. p.*	*P. a.*	*A. b.*	*E. c.*	*K. p.*	*P. a.*	*A. b.*
Penicillin G	7.15 ± 0.04	6.52 ± 0.29	6.71 ± 0.04	7.60 ± 0.20	4500 ± 200	2300 ± 1200	3500 ± 350	8700 ± 4000	N/A
Amoxicillin	6.68 ± 0.03	6.08 ± 0.21	6.22 ± 0.04	7.49 ± 0.18	1500 ± 100	750 ± 350	1050 ± 100	6500 ± 2500	None
Piperacillin	7.78 ± 0.05	7.84 ± 0.10	7.90 ± 0.03	7.78 ± 0.17	18 500 ± 2000	42 000 ± 8800	50 000 ± 3000	12 500 ± 5000	High
Mecillinam	4.31 ± 0.08	3.97 ± 0.34	4.55 ± 0.35	4.39 ± 0.24	6 ± 1	7 ± 4	30 ± 25	5 ± 3	None
Oxacillin	6.67 ± 0.07	6.71 ± 0.21	6.89 ± 0.07	7.17 ± 0.41	1400 ± 250	3300 ± 1350	5000 ± 800	3800 ± 2900	Low
Aztreonam	6.69 ± 0.07	8.13 ± 0.14	8.21 ± 0.03	8.14 ± 0.27	1500 ± 250	84 000 ± 2500	103 000 ± 7000	31 000 ± 21 000	None
Imipenem	6.59 ± 0.06	5.06 ± 0.24	5.44 ± 0.07	6.87 ± 0.23	1200 ± 150	75 ± 35	200 ± 30	1650 ± 850	Low/none
Ertapenem	7.49 ± 0.04	7.48 ± 0.14	7.52 ± 0.01	7.84 ± 0.13	9500 ± 800	18 500 ± 5600	22 000 ± 400	14 000 ± 4500	High
Doripenem	6.93 ± 0.11	6.25 ± 0.17	6.33 ± 0.02	7.68 ± 0.14	2600 ± 700	1100 ± 450	1500 ± 70	10 000 ± 3000	None
Biapenem	6.47 ± 0.16	4.31 ± 0.22	4.40 ± 0.10	6.64 ± 0.36	900 ± 300	13 ± 7	16 ± 4	1050 ± 650	None
Faropenem	6.22 ± 0.11	4.67 ± 0.23	4.87 ± 0.05	6.29 ± 0.38	500 ± 130	30 ± 20	50 ± 5	500 ± 400	Low
Ceftriaxone	7.70 ± 0.12	7.97 ± 0.09	7.96 ± 0.10	8.05 ± 0.19	16 000 ± 3800	57 000 ± 1200	59 500 ± 13 000	24 000 ± 11 000	Low
Cefoperazone	7.02 ± 0.06	8.17 ± 0.13	8.13 ± 0.11	8.41 ± 0.26	3200 ± 450	91 000 ± 2800	88 000 ± 22 000	59 000 ± 38 000	Low
Moxalactam	7.11 ± 0.10	7.54 ± 0.11	7.51 ± 0.07	8.02 ± 0.22	4000 ± 800	21 000 ± 6000	21 000 ± 3500	23 000 ± 12 000	Low
Meropenem	7.23 ± 0.03	6.93 ± 0.05	6.87 ± 0.05	7.91 ± 0.23	5600 ± 350	5000 ± 550	5000 ± 600	9500 ± 3000	N.A.

Interestingly, given that it is a monocyclic β-lactam, aztreonam was amongst the most potent of the PBP3 inhibitors tested. It did, however, show relatively low inhibition of *A. baumannii* PBP3 (*k*_inact_*/K*_I_ = 1500 ± 250 M^−1^ s^−1^) compared to the other tested PBP3s. The relatively low inhibition of the *A. baumannii* PBP3 by aztreonam has been observed previously and may reflect subtle differences in its active site compared to the tested PBP3s from other organisms.^11,12^ The cephalosporins tested (ceftriaxone and cefoperazone) manifested potent inhibition of all the PBP3s screened, as did the only tested oxacephem, moxalactam. Piperacillin shows potent inhibition of all the PBP3s, with weaker inhibition being observed for penicillin G, amoxicillin and oxacillin. Mecillinam, as expected,^19^ did not inhibit any of the tested PBP3s.

There were large variations in the level of inhibition observed for the carbapenems tested. Ertapenem showed potent inhibition of all the PBP3s, whereas meropenem had relatively low activity against *E. coli* and *K. pneumoniae* PBP3. Doripenem manifested potent activity against the *P. aeruginosa* PBP3 (*k*_inact_*/K*_I_ = 10 000 ± 3000 M^−1^ s^−1^), but had decreased activity against the other PBP3s, whilst biapenem, imipenem and faropenem were less active against all the tested PBP3s, with a notable drop in activity against the *E. coli* and *K. pneumoniae* PBP3s. Note that issues with solubility may impact on the results for imipenem.

Comparison of our results with reported *k*_inact_*/K*_I_ values, reveals good agreement for all the tested PBP3s. The *k*_inact_*/K*_I_ values reported by McLeod (using Bocillin™FL) for aztreonam (700 ± 100 M^−1^ s^−1^), imipenem (470 ± 50 M^−1^ s^−1^) and meropenem (5000 ± 1600 M^−1^ s^−1^) for *A. baumannii* PBP3,^20^ are in excellent agreement with our values (1500 ± 250 M^−1^ s^−1^, 1200 ± 150 M^−1^ s^−1^ and 5600 ± 350 M^−1^ s^−1^ respectively) with the same rank order of activity. There were slightly larger differences between our values and those measured by Shapiro *et al.* who reported values for both aztreonam (510 ± 40 M^−1^ s^−1^) and meropenem (1600 ± 200 M^−1^ s^−1^).^11^ These values also differ from those measured by McLeod *et al.*,^20^ despite the use of Bocillin™ FL by both McLeod *et al.*^20^ and Shapiro *et al.*^11^

Adam *et al.* employed a [^14^C]-benzylpenicillin radiolabelling competition assay to determine *k*_inact_*/K*_I_ values for *E. coli* PBP3 inhibition.^21^ Their results are in excellent agreement with the values measured here for penicillin G (4000 ± 200 M^−1^ s^−1^ compared to 2300 ± 1200 M^−1^ s^−1^), aztreonam (100 000 ± 10 000 M^−1^ s^−1^ compared to 84 000 ± 2500 M^−1^ s^−1^) and cefoperazone (80 000 ± 10 000 M^−1^ s^−1^ compared to 91 000 ± 2800 M^−1^ s^−1^), with only piperacillin differing (130 000 ± 15 000 M^−1^ s^−1^ compared to 42 000 ± 8800 M^−1^ s^−1^).^21^ Aztreonam, despite being amongst the most potent of the *E. coli* PBP3 inhibitors tested by us (along with cefoperazone), has a *k*_inact_*/K*_I_ that is several orders of magnitude lower than that measured by Shapiro *et al.* using a TAMRA-ampicillin probe (420 000 ± 50 000 M^−1^ s^−1^);^12^ this difference may be due to the limitation of the maximal value it is possible to measure with the different probes.

The *k*_inact_*/K*_I_ value measured for *P. aeruginosa* PBP3 for imipenem (1650 ± 850 M^−1^ s^−1^) is in good agreement with the literature value (Han *et al.*, 712 M^−1^ s^−1^).^22^ The *k*_inact_*/K*_I_ for meropenem with *P. aeruginosa* PBP3 (9500 ± 3000 M^−1^ s^−1^) is similar to our previously reported value (11 000 ± 3900 M^−1^ s^−1^).^23^ It is, however, 5-fold lower than that reported by Shapiro *et al.* (49 000 ± 2000 M^−1^ s^−1^) and Han *et al.* (63 760 M^−1^ s^−1^).^11,22^ Our value for aztreonam (31 000 ± 2100 M^−1^ s^−1^) is substantially lower than the reported values (296 000 ± 2000 M^−1^ s^−1^, 280 000 ± 40 000 M^−1^ s^−1^ and 4 396 400 M^−1^ s^−1^). As expected from literature comparison, however, aztreonam is identified in this work as the most potent inhibitor of *P. aeruginosa* PBP3.^11,12,22^

Our overall results with FC5 reflect previous work revealing the potency of cephalosporins for PBP3 inhibition, showing their breadth of activities across PBP3 from Gram-negative bacterial species.^24^ Carbapenems, whilst often being thought to principally inhibit PBP2 in Gram-negative bacteria,^12^ also demonstrate potency against the tested PBP3s (Table 1).

### Mass spectrometry competition experiments with *A. baumannii* PBP3

Protein observed mass spectrometry (MS) experiments were undertaken with *A. baumannii* PBP3 to complement the results from the fluorescence-based solution assays. Meropenem was used as a standard in competition assays, because it is readily available and potently inhibits *A. baumannii* PBP3. Meropenem and the subject inhibitor were combined in a 1 : 1 ratio (1 mM), added to *A. baumannii* PBP3 (100 µM) and, following a set incubation time, the reaction was quenched and analysed *via* protein observed electrospray ionisation MS (UPLC-ESI-Q-TOF-MS). The results correlate, although imperfectly, with those from measurements of *k*_inact_*/K*_I_ values using FC5. Note that upon MS analysis the meropenem acyl enzyme complex (AEC) fragments with a mass loss that under denaturing conditions may be caused by loss of the C3 carboxylic acid;^25^ the mass difference also corresponds to loss of the C6 hydroxyethyl sidechain.

Piperacillin and ertapenem both reacted to form PBP3 complexes in quantities exceeding that of meropenem, consistent with the *k*_inact_*/K*_I_ values (Table 1), whilst ceftriaxone reacted with similar efficiency as meropenem.

For compounds with *k*_inact_*/K*_I_ values similar to that of meropenem (penicillin G, cefoperazone and moxalactam, Table 1) mixtures of the test compound and meropenem complexes were observed. With penicillin G the similarities in molecular weight prevented differentiation from the meropenem complex. With both cefoperazone and moxalactam small peaks corresponding to the expected mass of the AEC were observed, with the meropenem adduct forming the major species (Table 1 and Table S1 and Fig. 2 and Fig. S10). With moxalactam only a very small AEC peak was observed for the adduct with an intact C3 group. The absence of a peak corresponding to loss of the C3 group with this inhibitor may be due to hydrolytic instability of the AEC. For cefoperazone, the AEC peak corresponds to the molecular weight anticipated following loss of the C3 group (Table 1 and Table S1 and Fig. S10–S12).

**Fig. 2:**
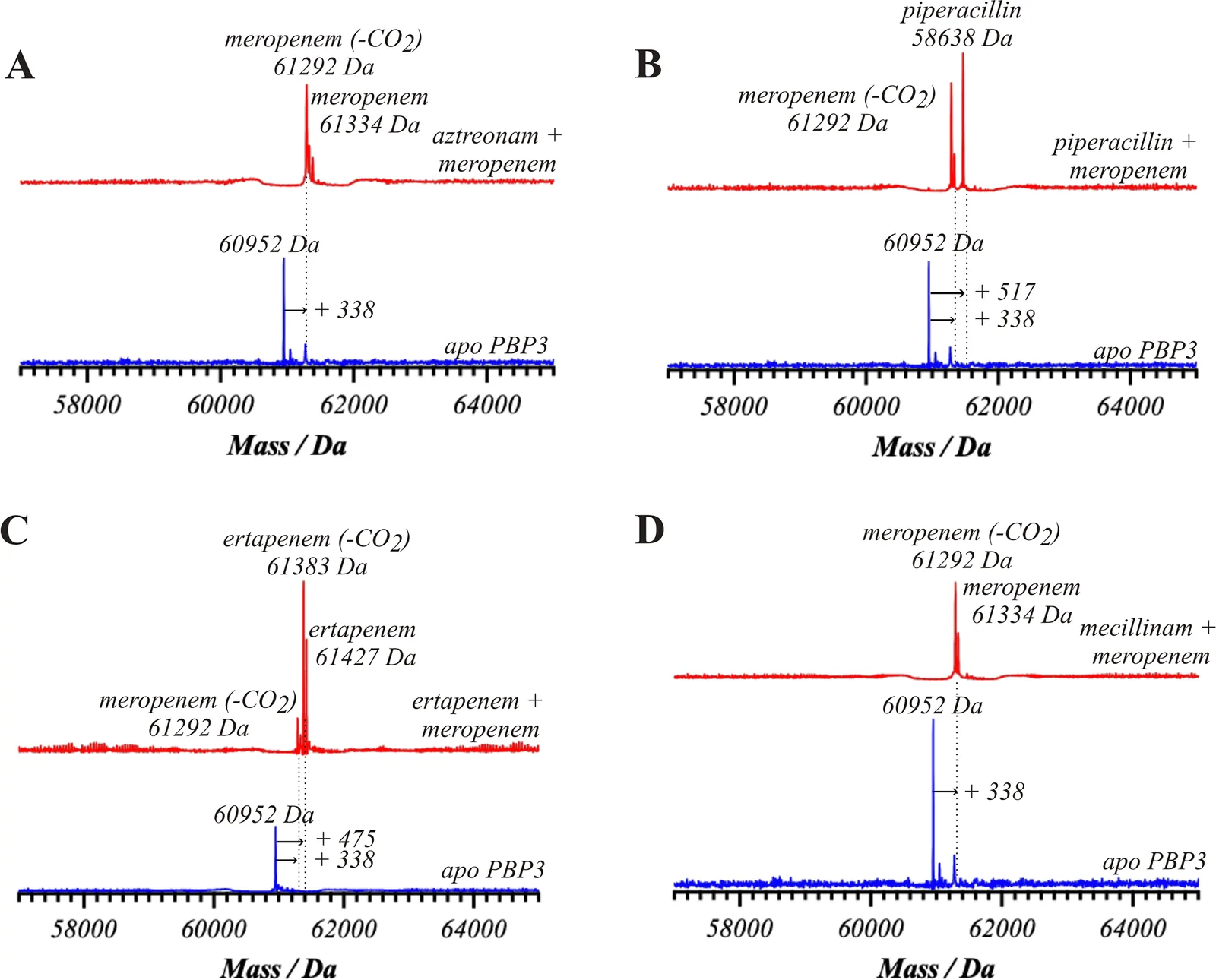
Protein observed mass spectra of *A. baumannii* PBP3 resulting from meropenem competition binding experiments inform on the rates of covalent reaction for different β-lactams compared to meropenem. (A) Meropenem outcompetes aztreonam; (B) piperacillin outcompetes meropenem; (C) ertapenem outcompetes meropenem; (D) meropenem outcompetes mecillinam. Observed and expected mass shifts are provided in Table S1. Additional mass spectra are provided in Fig. S10. See SI for method details. Note that the apparent loss of CO_2_ may, at least in part, be a result of the MS method;^25^ it is also possible that this mass shift is due to loss of the C6 hydroxyethyl sidechain.

For compounds with *k*_inact_*/K*_I_ values below that of meropenem (amoxicillin, mecillinam, oxacillin, aztreonam, imipenem, doripenem, biapenem and faropenem, Table 1) the meropenem complex was the major, or only, adduct species observed in all cases.

Whilst for the meropenem and oxacillin competition experiments the primary peak corresponds to the AEC formed with decarboxylated meropenem, the similarity in the molecular weights of the oxacillin and meropenem AECs, causes the second peak to comprise a mixture of both products (oxacillin (61 353 Da) and meropenem (61 336 Da, intact complex)). In the cases of imipenem and faropenem, a small additional peak corresponding to the imipenem/faropenem adduct compared to the major decarboxylated meropenem adduct peak (61 292 Da) was observed (Table 1 and Table S1 and Fig. S10).

Overall, the MS results correlate well with those observed with the FC5 assays. Compounds with pIC_50_ values for *A. baumannii* PBP3 that were similar to, or which exceeded that of meropenem, gave rise to MS adducts for either the test compound only, or a mixture of the test compound and meropenem. Compounds with similar or slightly lower pIC_50_s compared to meropenem give rise to mixed MS adducts, where the meropenem product either predominated, or was the sole adduct observed, whilst for compounds with low pIC_50_ values only the meropenem adduct was observed (Table 1 and Table S1 and Fig. 2 and Fig. S10, S12).

### Crystallography

To investigate whether the PBP3 binding modes of carbapenems with different C2 groups correlate with their rates of reaction, crystal structures of PBP3 in complex with doripenem, biapenem and ertapenem were obtained. The structures were solved (using an AlphaFold model as the search model) in the *P*2_1_2_1_2_1_ space group with one molecule per asymmetric unit and diffracted to 1.6 Å (doripenem and ertapenem) and 1.9 Å (biapenem) resolution. As reported, the *P. aeruginosa* PBP3 fold is comprised of two domains; a C-terminal transpeptidase catalytic domain and an N-terminal dimerization domain that enables interactions with other proteins within the divisome complex.^23^

As with reported structures for imipenem and meropenem, in each case the carbapenems react with the nucleophilic serine of *P. aeruginosa* PBP3 (Ser294) to form an AEC (Fig. 3), wherein the β-lactam derived carbonyl oxygen is positioned to form a hydrogen bond with the Thr487 backbone NH (2.9–3.0 Å).^22^ As reported, the C3 carboxylic acid is positioned to form a salt bridge interaction with Lys484 (4.4–4.7 Å) and a hydrogen bond with the Gly534 backbone NH (3.7–3.9 Å; the latter interaction is water mediated in the biapenem structure).^22^ Additional carboxylic acid interactions varied across the AEC structures; with the doripenem derived AEC there is an additional water mediated interaction with Lys484. The ertapenem carboxylic acid is positioned to form a hydrogen bond with the sidechain hydroxyl of Thr487 (2.8 Å) and a water mediated interaction with Gly535. The biapenem carboxylic acid also forms an additional water mediated interaction with Gly535.

**Fig. 3:**
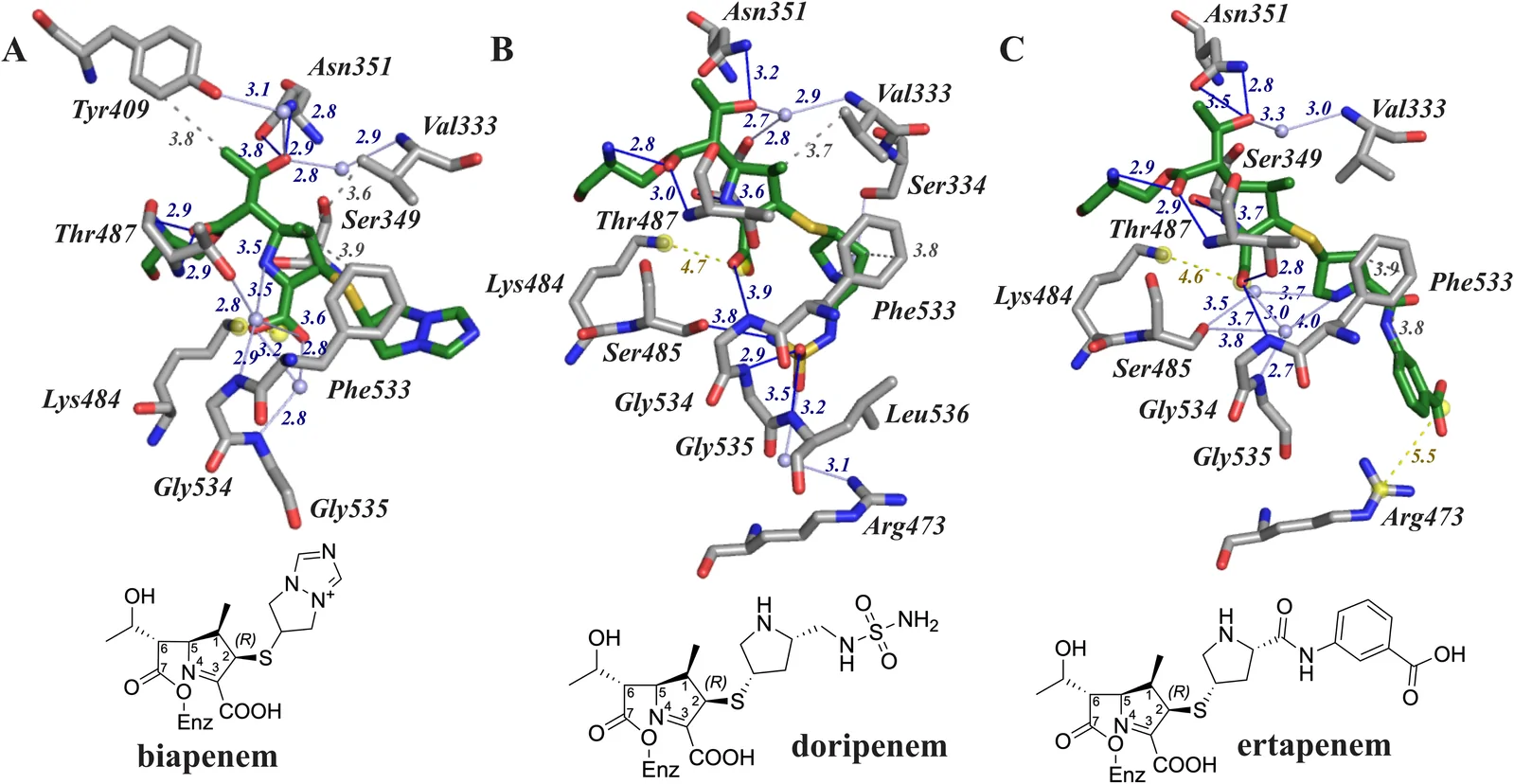
Crystallographic studies reveal key interactions of carbapenems with *P. aeruginosa* PBP3; (A) biapenem (weak PBP3 inhibitor), (B) doripenem (intermediate potency PBP3 inhibitor), and (C) ertapenem (potent PBP3 inhibitor) interact differently with the active site due to their different C2 groups. The C2 group of biapenem does not form any interactions, the doripenem sulfamide forms three hydrogen bond interactions, whilst the terminal carbonyl of the ertapenem C2 group forms a salt bridge and its phenyl group forms a hydrophobic interaction. The AECs were refined in their imine forms with an sp3 C2 carbon (2*R*-stereochemistry). Structures are deposited with the following PDB codes: 33GK, 33GL, 33GO. Although the AECs were modelled with the imine form, it cannot be ruled out the enamine or (2*R*)-imine tautomeric forms are relevant in solution.

In the three new structures, the C6-hydroxyethyl carbapenem sidechain is positioned to form a hydrogen bond interaction with Asn351 (2.9–3.1 Å) and a water-mediated interaction with the Val353 backbone amide NH (2.8–3.3 Å) (Fig. 3 and Fig. S13–S15). The doripenem C6 hydroxyethyl sidechain is positioned to form an additional water mediated hydrogen bonding interaction with Ser349 sidechain alcohol (Fig. 3B).

It is important to note that, whilst it is possible that either the imine or the enamine form of the bound carbapenem may occur on formation of the AEC, the imine was modelled in our structures, as this improved refinement statistics. It is, however, plausible that a mixture of tautomers is present, including in solution.^26,27^ The imine nitrogen of the AEC 5-membered ring forms a hydrogen bond with the backbone carbonyl oxygen of Ser349 (3.5–3.7 Å), whilst the C1 methyl sidechain forms a hydrophobic interaction with the sidechain of Val333 (3.6–3.7 Å), though this is absent in the case of the ertapenem derived AEC.

The main variation between the binding modes of the carbapenems relates to their C2 side chains. Additional active site interactions occur with the doripenem and ertapenem AECs compared to biapenem, for which no direct interactions with the shorter C2 side chain were observed.

The terminal NH_2_ of the doripenem AEC C2 sulfamide group is positioned to form a hydrogen bond with the Ser485 backbone carbonyl oxygen (Fig. 3C and Fig. S14). One of the oxygen atoms of the doripenem AEC sulfamide group is positioned to form additional interactions with Gly535 (backbone NH) and Leu536 (backbone NH). The other sulfamide oxygen atom is positioned to form a water mediated interaction with Arg473 (sidechain NH). Similarly, the terminal carbonyl of the ertapenem AEC carboxyphenyl group forms a salt bridge with the sidechain of Arg473 (Fig. 3C and Fig. S15). Note that the density for the terminal portion of the C2 groups in the doripenem and ertapenem derived structures is weaker than the rest of the ligand, likely reflecting conformational mobility (Fig. S16). Indeed, the ertapenem C2 group may adopt two conformations within the active site, forming an additional set of interactions. The C2 group of doripenem may also adopt more than one conformation, whilst the pyrazolotriazole group of biapenem is apparently more rigid, sitting in a single conformation in the PBP3 active site.

Comparison with reported structures of *P. aeruginosa* PBP3 in complex with meropenem and imipenem provides additional insight on how the differences in the C1 and C2 functional groups affects the binding (SI Table S4) and activity of these compounds.^22^ Meropenem, whilst reacting most rapidly of the five carbapenems investigated with *P. aeruginosa* PBP3, has a similar pIC_50_ to doripenem and ertapenem. The meropenem AEC C2 sidechain forms four water-mediated interactions (pyrrolidine NH to sidechain OH of Ser485, carbonyl to backbone NH of Gly535, OH of Ser485 and to internal meropenem carboxylate group) (Fig. S17). Imipenem has a linear 2-(aminomethylideneamino)ethylsulfanyl C2 group positioned to form two hydrogen bond interactions with Ser334 (imine N to sidechain OH and water mediated to backbone NH) and one with Gly469 (amine NH to backbone carbonyl).

## Conclusions

We have developed an efficient fluorescence-based assay for measurement of PBP3 inhibition employing the cephalosporin compound FC5 (Fig. 1), a probe previously applied for measurement of β-lactamase inhibition.^15^ This new method enables rapid determination of *k*_inact_/*K*_I_ values for many PBPs and compounds from endpoint IC_50_ measurements. This assay and the associated methodology, derived from prior work by Miyahisa *et al.*, should be broadly applicable for PBPs from other organisms.^17^ The results from the FC5 method were validated *via* complementary competition mass spectrometry experiments measuring the extent of covalent reaction with the *A. baumanii* PBP3.

Interestingly, the results of *in vitro* inhibition experiments demonstrated that aztreonam, the only globally used monobactam, was the most potent inhibitor of all the PBP3s tested, except for the *A. baumannii* PBP3. The cephalosporins were the second most potent inhibitors, with carbapenems and penicillins showing reduced potency. Ertapenem and meropenem did, however, show high potency against all the enzymes tested, indicating that carbapenems can be potent PBP3 inhibitors.

Carbapenems have often been perceived to function predominantly *via* PBP2 inhibition; however, some carbapenems are also potent PBP3 inhibitors.^28^ X-ray crystallographic analyses provide additional information on how subtle changes in the functional groups at the carbapenem C2 position within these compounds have a large impact on their inhibitory activity. The order of reactivity in solution correlates with the number of hydrogen bond interactions formed with ertapenem and doripenem, which are both potent inhibitors forming 8 and 9 hydrogen bonds, respectively, with PBP3; biapenem, a weaker inhibitor, is observed to form only 5 hydrogen bonds with PBP3 (Table S3). It is, however, important to consider that the AECs observed crystallographically may not reflect the preferred initial, pre-acylation binding poses. The different C2 sidechains may affect the reactivity of the compounds; reduced flexibility may slow entry into the active site and hence covalent reaction, perhaps contributing to the decreased pIC_50_ for biapenem compared to those measured for doripenem and ertapenem with *P. aeruginosa* PBP3.

It is important to note that there may well be differences in PBP inhibitor engagement between studies with isolated PBPs and the cellular context. In cells, multiple PBPs may be accessible to a single inhibitor and PBP-inhibitor complex interactions with substrates may also be important. We envisage that our assay could be readily incorporated into a PBP inhibitor development platform following initial experiments with membrane preparations that containing all the PBPs in the bacteria, to identify specific set of PBPs for which optimisation with isolated PBPs would be valuable.

The new solution assays and accompanying crystallography and mass spectrometry information will help guide development of new PBP inhibitors. The simultaneous inhibition of multiple PBPs is likely to be advantageous for achieving rapid cell death. The crystallographic information provided here, alongside that gathered from the *in vitro* assays, will inform on the development of both standalone PBP3 inhibitors and more potent, dual functional PBP2 and PBP3 inhibitor compounds for use as antibacterials.

## Author contributions

H. G. S. conceptualisation, methodology, analysis, investigation, writing. I. E. T. production of FC5 probe. S. A. M. analysis, editing. A. J. M. F. conceptualisation, C. J. S. conceptualisation, methodology, analysis, supervision, writing.

## Conflicts of interest

There are no conflicts to declare.

## Supplementary Material

CB-OLF-D6CB00304D-s001

## Data Availability

Crystallographic data has been deposited at the protein data bank (PDB) under the accession codes: 33GK, 33GL and 33GO. Additional figures, tables and source data can be found in the supplementary information (SI). Supplementary information is available. See DOI: https://doi.org/10.1039/d6cb00304d.
