# Supplementary material for: Efficient assays for measuring inhibition of penicillin binding protein 3 (PBP3) transpeptidases
Source: RSC Chem Biol. 2026 Nov 10. Online ahead of print. doi: 10.1039/d6cb00304d (PMC13749169; doi:10.1039/d6cb00304d)
Supplement: Supplementary file 1 [file CB-OLF-D6CB00304D-s001.pdf]

Supplementary Information (SI) for RSC Chemical Biology.
This journal is © The Royal Society of Chemistry

Supplementary Information

Efficient Assays for Measuring Inhibition of Penicillin Binding Protein 3 (PBP3) Transpeptidases

Helen G. Smith,[a,b*] Ignacio E. Tobal,[a,b] Stephen A. Marshall,[a,b] Alistair J.M. Farley,[a,b] Christopher J. Schofield.[a,b*]

[a] Department of Chemistry, University of Oxford, 12 Mansfield Road, Oxford, OX1 3TA, UK
[b] Ineos Oxford Institute for Antimicrobial Research, University of Oxford, 12 Mansfield Road, Oxford, OX1 3TA, United Kingdom.
* Corresponding authors.

Contents

1. Materials and Methods 3
1.1 Synthesis 3
1.2 Biochemistry 3
1.2.1 Generation of plasmids encoding PBPs 3
1.2.2 Recombinant protein production and purification 3
1.2.3 pIC_{50} and k_{inact}/K_I measurements 4
1.2.4 Mass spectrometry competition experiments 5
1.2.5 Protein crystallography 6

2. Supplementary Figures and Tables
Figure S1: SDS-PAGE and deconvoluted mass spectra of PBP3 proteins 7
Figure S2: Reaction scheme for reversible probe-competition method 8
Figure S3: Z'-factor calculation for PBP3 proteins 9
Figure S4: Determination of k_{obs} for PBP3 proteins 10
Figure S5: Measurement of $(k_{inact}/K_I)_{probe}$ for PBP3 proteins 11
Figure S6: Determination of pIC_{50} and k_{inact}/K_I for *A. baumannii* PBP3 12
Figure S7: Determination of pIC_{50} and k_{inact}/K_I for *E. coli* PBP3 13
Figure S8: Determination of pIC_{50} and k_{inact}/K_I for *K. pneumoniae* PBP3 14
Figure S9: Determination of pIC_{50} and k_{inact}/K_I for *P. aeruginosa* PBP3 15
Figure S10: Competition mass spectrometry experiments with *A. baumannii* PBP3 16
Table S1: Competition mass spectrometry experiment analysis 17
Figure S11: Structures of compounds utilised in this work 18
Figure S12: Proposed mechanism for binding of cephalosporins to PBP3 19
Table S2: Crystallography data collection and refinement statistics 20
Figure S14: LigPlot+ interaction map of *P. aeruginosa* PBP3 biapenem complex 21
Figure S15: LigPlot+ interaction map of *P. aeruginosa* PBP3 doripenem complex 22
Figure S16: LigPlot+ interaction map of *P. aeruginosa* PBP3 ertapenem complex 23
Figure S17: Polder OMIT maps showing crystallographic acyl-enzyme complexes 24
Figure S18: Interactions present in reported structures of carbapenems with PBP3 25
Table S3: Interactions formed by different inhibitors in complex with PBP3 26

3. References 27

Materials and Methods

Synthesis

The FC5 probe was synthesised according to the reported procedure.[1]

Biochemistry

Generation of plasmids encoding PBPs

Unless otherwise stated, plasmids encoding for PBP3s were from GenScript, UK. In each case the gene was truncated to remove the *N*-terminal transmembrane helix; residue numbers for constructs are given below. DNA encoding for *K. pneumoniae* PBP3 (GenBank: WP_105424109.1, residues 71 to 570) was inserted into the pET-28a(+) vector between the NcoI and XhoI restriction sites. The DNA encodes for *K. pneumoniae* PBP3 with an N-terminal His_6-tag with a thrombin cleavage site (sequence: MGSSHHHHHHSSGLVPRGSTS). DNA encoding for *A. baumannii* PBP3 (GenBank: WP_008941134, residues 64 to 609) was inserted into the pET-24a(+) vector between the NdeI and XhoI sites, to encode for a protein with an N-terminal His_6-tag. The *P. aeruginosa* PBP3 producing plasmid was prepared as reported.[2] Thus, DNA encoding for PBP3 from *P. aeruginosa* PAO1 (residues 50 to 579) with an N-terminal His_6-tag was inserted into the pET-28a vector between the NcoI and HindIII sites.[2] DNA encoding for *E. coli* PBP3 (residues 49 to 588) was amplified from New England Biolabs (NEB) BL21(DE3) genomic DNA and inserted into the pET-28a vector between HindIII and XhoI restriction sites, to encode for a protein with an N-terminal His_6-tag and thrombin cleavage site, using the following primers; *EcPBP3-FP ATTAAGCTTCCCCGGATATGC TGGTGAAAGAGG, EcPBP3-RP ATACTCGAGTTACGATCTGCCACCTGTCCCCTC*. PCR was performed using the NEB Q5 Hot Start 2X Master Mix (according to the manufacturer's protocol). PCR product purification was performed using the Thermo Fisher Scientific GeneJET DNA Extraction Kit (according to the manufacturer's instructions); the final elution step was modified to elute the DNA into water (30 µL). The PCR product was digested using HindIII-HF and XhoI (NEB, 3 hours, 37 °C). Following digestion, the product was purified using a Thermo Fisher Scientific GeneJET DNA Extraction Kit according to the manufacturer's instructions; the final elution step was modified to elute the DNA into water (30 µL). An identical procedure was followed to digest pET-28a. Following purification, samples were ligated using the T4 DNA ligase (NEB) according to the manufacturer's instructions. Samples were ligated at room temperature overnight.

Recombinant protein production and purification

P. aeruginosa PBP3 was purified as reported.[2] In brief, BL21(DE3) cells (NEB) were transformed with the pET-28a(+) vector containing the *P. aeruginosa* PBP3 gene via heat shock (42 °C, 45 seconds). Following addition of 2xYT media (Formedium, 900 µL) the mixture was incubated (1 hour, 37 °C) and subsequently plated onto 2xYT agar (50 µg/mL kanamycin), then incubated (overnight, 37 °C). A single colony was selected and grown overnight in 2xYT medium (100 mL, 37 °C, 200 rpm, 50 µg/mL kanamycin). Terrific broth (TB) autoinduction medium (Formedium, 6 L, 50 µg/mL kanamycin) was inoculated with the preculture (6 mL per 1 L of TB) and incubated overnight (24 hours, 25 °C, 180 rpm). Cells were harvested via centrifugation (8,000 rpm, 10 minutes) and stored at –80 °C.

Cells were thawed and resuspended in buffer (25 mM Tris, 400 mM NaCl, 10 mM imidazole, 10 $\%_{v/v}$ glycerol, pH 8.0, 0.1 $\%_{v/v}$ CHAPS, 100 µg Roche DNaseI, 1 cOmplete mini EDTA-free protease inhibitor tablet, Roche). Cells were lysed via cell disruption (25 kPsi) and cell debris pelleted via centrifugation (22,000 rpm, 30 minutes, 4 °C). The supernatant was filtered (0.45 µm) and loaded onto a HisTrap column (5 mL, Cytiva, flow rate 2.5 mL/min). The column was washed with resuspension buffer (15 CV); the protein was eluted with a stepwise

gradient of imidazole; 50 mM (5 CV), 100 mM (3 CV), 200 mM (3 CV), 300 mM (3 CV), 500 mM (3 CV). Gel electrophoresis (180 V, 45 mins) was carried out using a 4 – 12 % NuPAGE gel (Invitrogen) to identify fractions containing sufficiently purified *P. aeruginosa* PBP3. These were pooled, concentrated and buffer exchanged using a PD-10 column (Cytiva) into buffer containing 25 mM Tris, 400 mM NaCl, 10%$_{v/v}$ glycerol, pH 8.0.

A. baumannii PBP3 was overproduced and purified according to a reported procedure.[3] In brief, following growth in autoinduction media (6 L, 25 °C, 140 rpm for 24 hours), cells were harvested via centrifugation (8,000 rpm, 4 °C, 10 minutes) and stored at – 80 °C. Cells were thawed and resuspended in buffer (25 mM Tris, 400 mM NaCl, 10 mM imidazole, 10 %$_{v/v}$ glycerol, pH 8.0, 0.1%$_{v/v}$ CHAPS (3-[(3-cholamidopropyl)dimethylammonio]-1-propanesulfonate) buffer, 100 μg DNaseI, 1 cOmplete EDTA free protease inhibitor tablet) and lysed via cell disruptor (25 kPsi). The cell debris was pelleted via centrifugation (20,000 rpm, 30 minutes, 4 °C); the supernatant was filtered (0.45 μm filter) and loaded onto a 5 mL Ni-NTA column (GE Healthcare) that had been pre-equilibrated in buffer (25 mM Tris, 10 mM imidazole, 400 mM NaCl, 10 %$_{v/v}$ glycerol, pH 8.0) at a flow rate of 2.5 mL/min. The column was washed with the resuspension buffer (75 mL) and the protein eluted with a stepwise gradient of imidazole; 50 mM (25 mL), 100 mM (15 mL), 200 mM (15 mL), 300 mM (15 mL), 500 mM (25 mL). Gel electrophoresis (180 V, 45 mins) was carried out using a 4-12 % NuPAGE gel (Invitrogen) to identify fractions containing sufficiently purified *A. baumannii* PBP3. These were pooled, concentrated and buffer exchanged via PD-10 column (Cytiva) into buffer containing 25 mM Tris, 200 mM NaCl, 10%$_{v/v}$ glycerol, pH 8.0.

K. pneumoniae PBP3 was purified following the same procedure to that used for *A. baumannii* PBP3.

E. coli PBP3 was produced using LB-Broth Miller media (Formedium). Following transformation of BL21(DE3) cells (NEB) and production of an overnight culture, LB media were inoculated (6L, 6 mL of preculture, 50 μg/mL kanamycin) and incubated (37 °C, 180 rpm) until an optical density at 600 nm (OD_{600}) of 0.6 was reached. Isopropyl β-D-1-thiogalactopyranoside (IPTG) was added to a final concentration of 1 mM and the cultures incubated overnight (20 °C, 180 rpm). The cells were pelleted and stored at -80 °C. The cells were resuspended in buffer (20 mM Tris pH 7.5, 300 mM NaCl, 20 mM imidazole, 1 Roche cOmplete protease inhibitor tablet, 100 μg Roche DNase I) and lysed using a cell disruptor (20 kPsi). The cell debris was pelleted (20,000 rpm, 4 °C, 30 minutes), the supernatant was filtered (0.45 μm) and loaded onto a 5 mL Ni-NTA column (GE Healthcarre) pre-equilibrated in resuspension buffer. The column was eluted stepwise with increasing concentrations of the resuspension buffer supplemented with imidazole; 20 mM (75 mL), 50 mM (25 mL), 100 mM (15 mL), 200 mM (15 mL), 300 mM (15 mL), 500 mM (25 mL). Gel electrophoresis (180 V, 45 mins) was carried out using a 4-12 % NuPAGE gel (Invitrogen) to identify fractions containing sufficiently purified *E. coli* PBP3. Fractions containing sufficiently purified *E. coli* PBP3 were pooled and buffer exchanged (10 mM Tris pH 7.5, 200 mM NaCl, 20 % glycerol). The purities and sequence identities of all proteins were measured via SDS-PAGE and intact protein mass spectrometry (Supplementary Figure S1).

pIC_{50} and k_{inact}/K_I measurements

A final assay volume of 25 μL was used. To each well of a 384-well black, non-binding microtiter plate (Greiner Bio-One) were added FC5.[1] (12 μL, 1.041 μM stock concentration, 500 nM final concentration) and the appropriate inhibitor (0.5 μL, serial dilution in DMSO; 3-fold dilution series from top concentration 100 μM to 5 nM). The requisite PBP3 (12.5 μL, 100 nM stock concentration, 50 nM final concentration) was added to initiate reaction. For positive control wells the inhibitor was replaced with DMSO; for negative control wells, both the

protein and inhibitor were replaced with buffer. The assay buffer used was 50 mM potassium phosphate pH 7.4. Following incubation at room temperature (1.5 hours *K. pneumoniae* PBP3, 2 hours *E. coli* PBP3, 3 hours *A. baumannii* PBP3, 3.5 hours *P. aeruginosa* PBP3) the fluorescence (λ_{ex} = 380 nm, λ_{em} = 460 nm) was measured using a BMG Labtech CLARIOstar plate reader. Data points were normalised between the positive and negative controls. The normalised % response was plotted as a function of $\log_{10}$([inhibitor]) and the IC_{50}^{app} was determined by fitting the data to the following equation: $Y = 100 / (1+10^{(-LogIC50+X)})$ where Y = % response and X = $\log_{10}$([inhibitor]).[4]

To calculate the second order rate constant k_{inact}/K_I the method of de Munnik *et al.* was applied.[4,5] Initially, the k_{inact}/K_I of the FC5 probe was calculated by measurement of the fluorescence intensity of the reaction between each PBP (50 nM) and FC5 (two-fold dilution series from 1 to 0.078125 µM, for *P. aeruginosa* PBP3 a two-in-three dilution series from 2 to 0.012 µM was used) in 50 mM sodium phosphate pH 7.4.The increase in fluorescence intensity increase was monitored using a BMG Labtech CLARIOstar plate reader (λ_{ex} = 380 nm, λ_{em} = 460 nm). The observed kinetic rate constant (k_{obs}) was calculated using the following equation [4]:

$$Y = (Y_\infty - Y_0)\left[1 - e^{(-k_{\mathrm{obs}}*\mathrm{t})}\right] + Y_0$$

The $(k_{inact}/K_I)_{probe}$ was calculated using the following equation [4]:

$$k_{\mathrm{obs}} = (k_{\mathrm{inact}}/K_{\mathrm{I}})_{\mathrm{probe}}[\mathrm{probe}]$$

The half-life ($t_{1/2}$) of the probe at 500 nM was determined using the following equation [4]:

$$t_{1/2} = \frac{\ln 2}{k_{\mathrm{obs}}}$$

The incubation time to achieve > 95 % receptor occupancy was calculated. A lower occupancy of > 90 % was used for *P. aeruginosa* PBP3 due to the longer $t_{1/2}$ observed for it. The values of k_{obs} and $t_{1/2}$ for each PBP are given in Table 1. The $(k_{inact}/K_I)_{inhibitor}$ values for each inhibitor were calculated using the following equation [4]:

$$\frac{k_{\mathrm{inact}}}{K_{\mathrm{I}}} = \frac{(k_{\mathrm{inact}}/K_{\mathrm{I}})_{\mathrm{probe}}[FC5]}{\mathrm{IC}_{50}}$$

Mass spectrometry competition experiments

A. baumannii PBP3 (100 µM) in ammonium acetate (50 mM, pH 7.5) was preincubated with a 1:1 mixture of the inhibitor of interest (1 mM) and meropenem (1 mM) for ten minutes prior to quenching by addition of formic acid (final volume:1 $\%_{v/v}$). Samples were diluted to a final concentration of 15 µM prior to analysis with a Waters XeVo G2-s mass spectrometer coupled with a Waters Acquity-UPLC. 5 µL samples were injected onto a ThermoFisher Scientific ProSwift RP-4H (1mm x 50 mm) column pre-equilibrated in 95 $\%_{v/v}$ water, 5 $\%_{v/v}$ MeCN. The column was eluted via a linear gradient from 5 to 95 $\%_{v/v}$ MeCN in water over ten minutes. All solvents contained 0.1 $\%_{v/v}$ formic acid. Data were analysed using MassLynx Version 4.1. The mass spectra obtained were deconvoluted using the MaxEnt1 algorithm.

Protein crystallography

Crystals of *P. aeruginosa* PBP3 in complex with known PBP3 inhibitors (doripenem, ertapenem, and biapenem) were obtained and analysed as reported.[2] In brief a truncated crystallisation construct of *P. aeruginosa* PBP3 (4 mg/mL, 10 mM Tris pH 8.0, 100 mM NaCl) was incubated with inhibitor (0.5 mM) for five minutes prior to setup of crystal trays. Crystals were achieved in several conditions; conditions SG1 C10 (Molecular Dimensions), SG1 B3 and PACT C4 (Molecular Dimensions) were used to obtain datasets.

X-ray data were collected at beamline I03 at Diamond Light Source (Harwell, UK). Diffraction data were indexed and integrated using DIALS.[6] Crystals were in the $P2_12_12_1$ space group with one molecule in each asymmetric unit. Each structure was solved via molecular replacement using Phaser.[7] A model of *P. aeruginosa* PBP3 (PDB 9FZ8) was used as the search model.[8] Multiple rounds of refinement were undertaken using PHENIX with manual model building undertaken using COOT.[9,10] This process was repeated until R_{free} and R_{work} converged. The statistics for the final, refined structures are provided (Table S2). Ligand restrains files were generated in AceDRG in the CCP4 suite of programs.[11–13]

The structures are deposited within the Protein Data Bank with accession codes: 33GL, 33GK, 33GO.

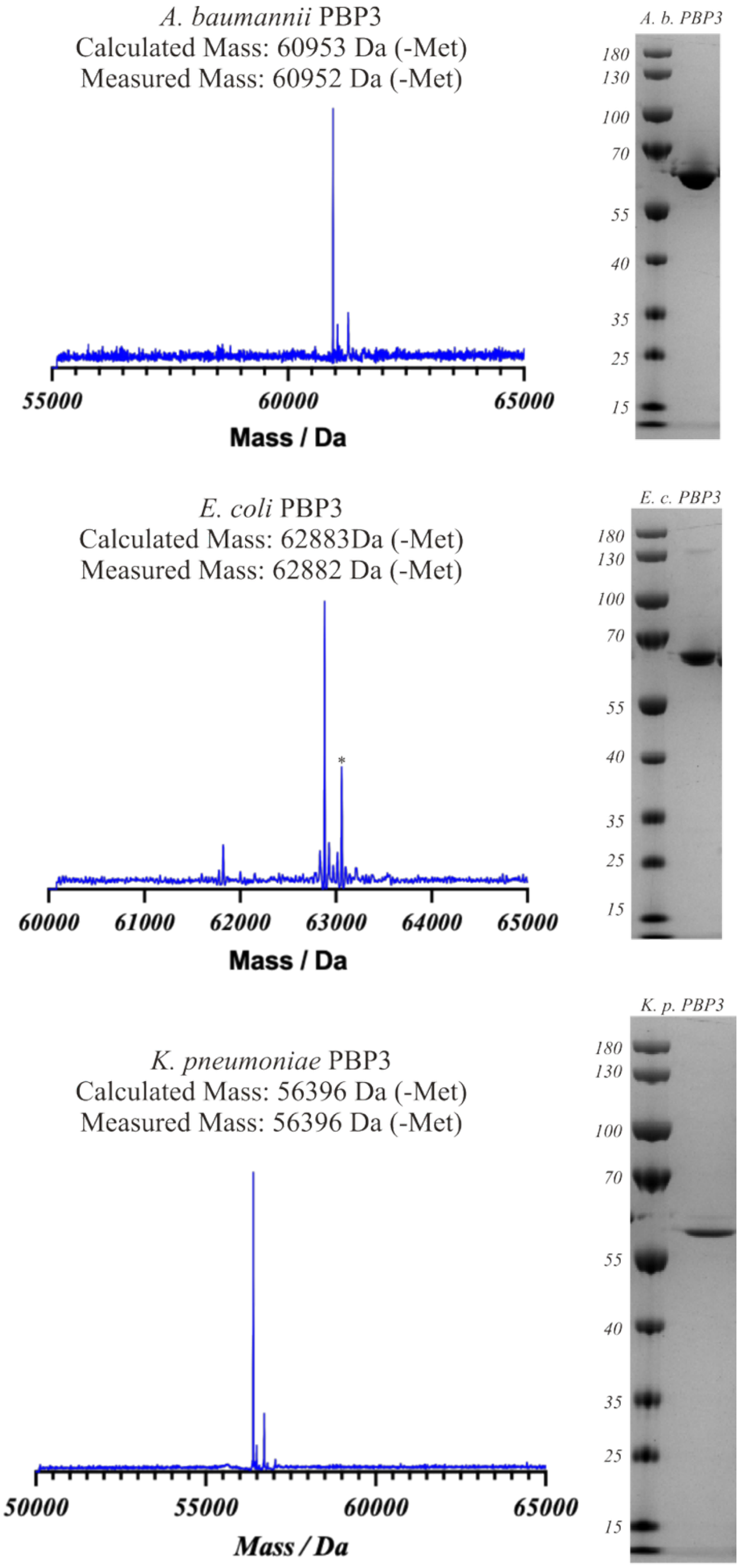

Supplementary Figure S1: SDS-PAGE gel and deconvoluted mass spectra of *A. baumannii* PBP3, *E. coli* PBP3 and *K. pneumoniae* PBP3. *Indicates post-translational gluconylation (+ 178 Da).[14] See Materials and Methods section for experimental details.

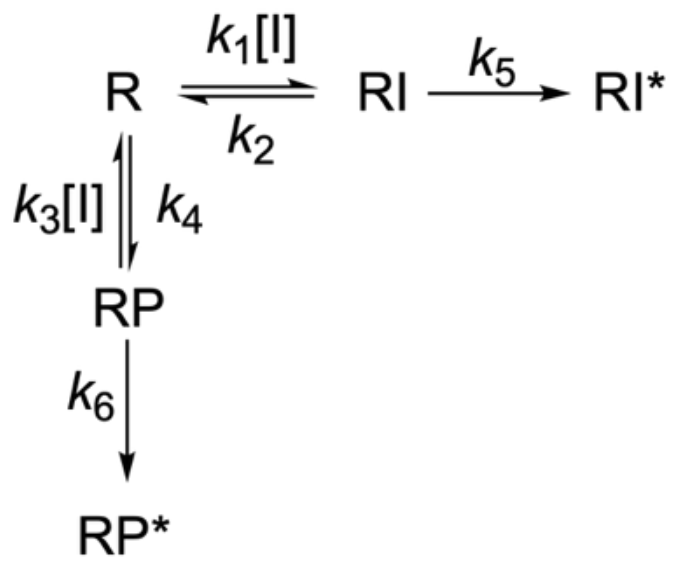

Supplementary Figure S2: Reaction scheme for the reversible probe-competition method employed by Miyahisa *et al*.[5] where R = receptor, I = inhibitor and P = probe.

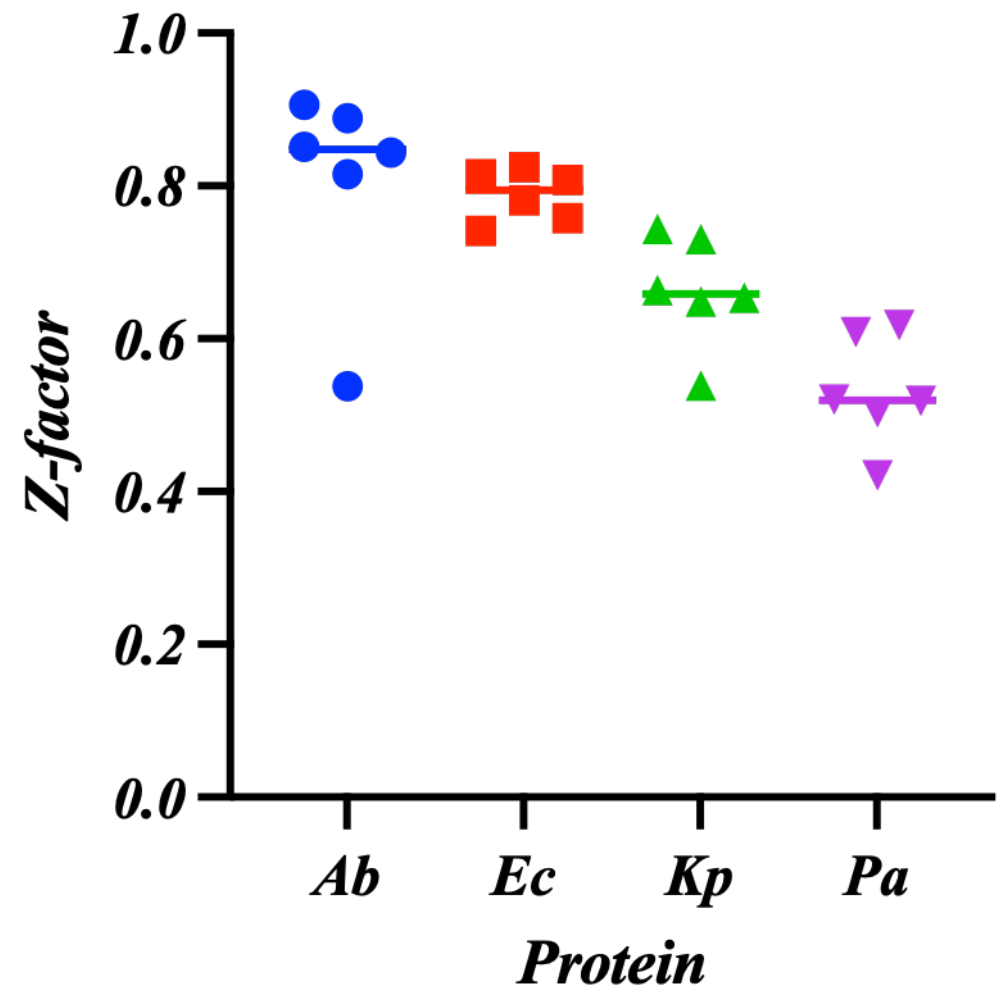

Supplementary Figure S3: Z′-factor calculation for *A. baumannii* PBP3 (Ab), *E. coli* (Ec) PBP3, *K. pneumoniae* (Kp) and *P. aeruginosa* PBP3 (Pa). FC5[1] (500 nM) and the requisite PBP3 (50 nM) were reacted in 50 mM potassium phosphate buffer, pH 7.4. See Materials and Methods for assay details.

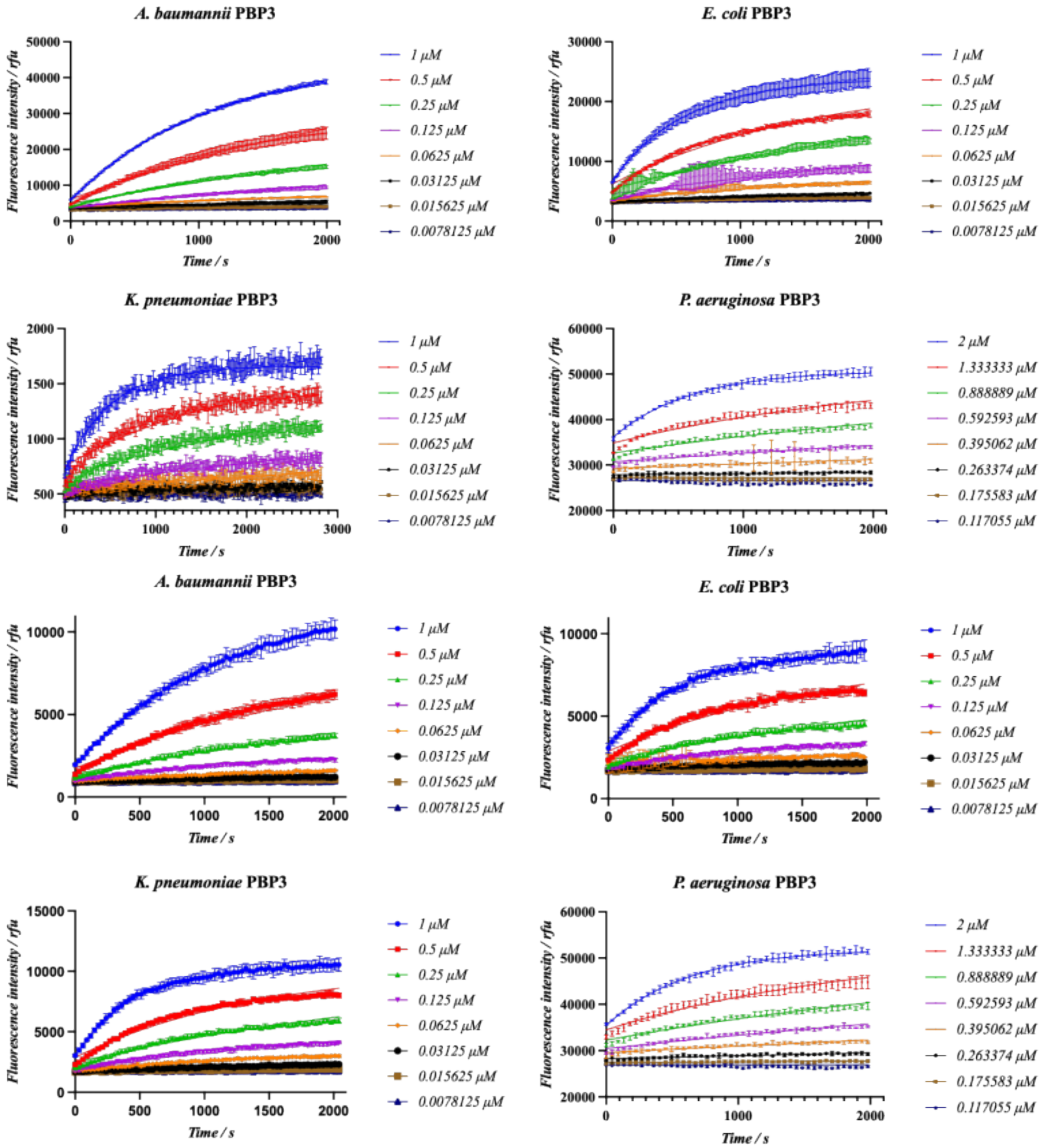

Supplementary Figure S4: Determination of the observed kinetic rate constant (k_{obs}) using varied concentrations of FC5[1] and a fixed concentration of the shown PBP3 (50 nM) in 50 mM potassium phosphate buffer, pH 7.4. Two data sets are shown for each PBP3. See Materials and Methods for assay details.

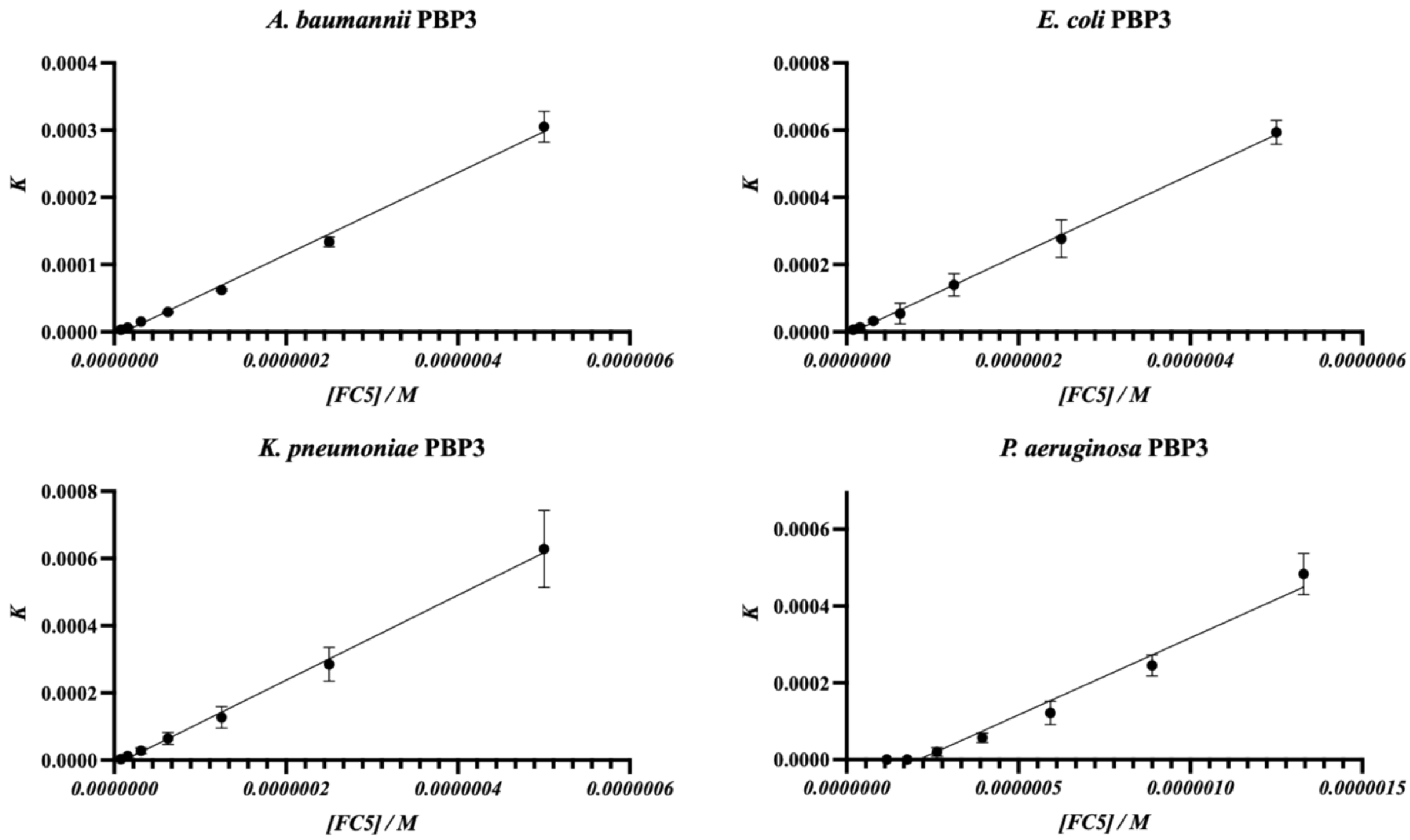

Supplementary Figure S5: Measurement of the observed kinetic rate constant (k_{obs}) enables calculation of the second order rate constant ($(k_{inact}/K_I)_{probe}$) for the different PBP3s. Error bars represent standard deviations of two repeats, each comprised of technical triplicates. See Materials and Methods for assay details.

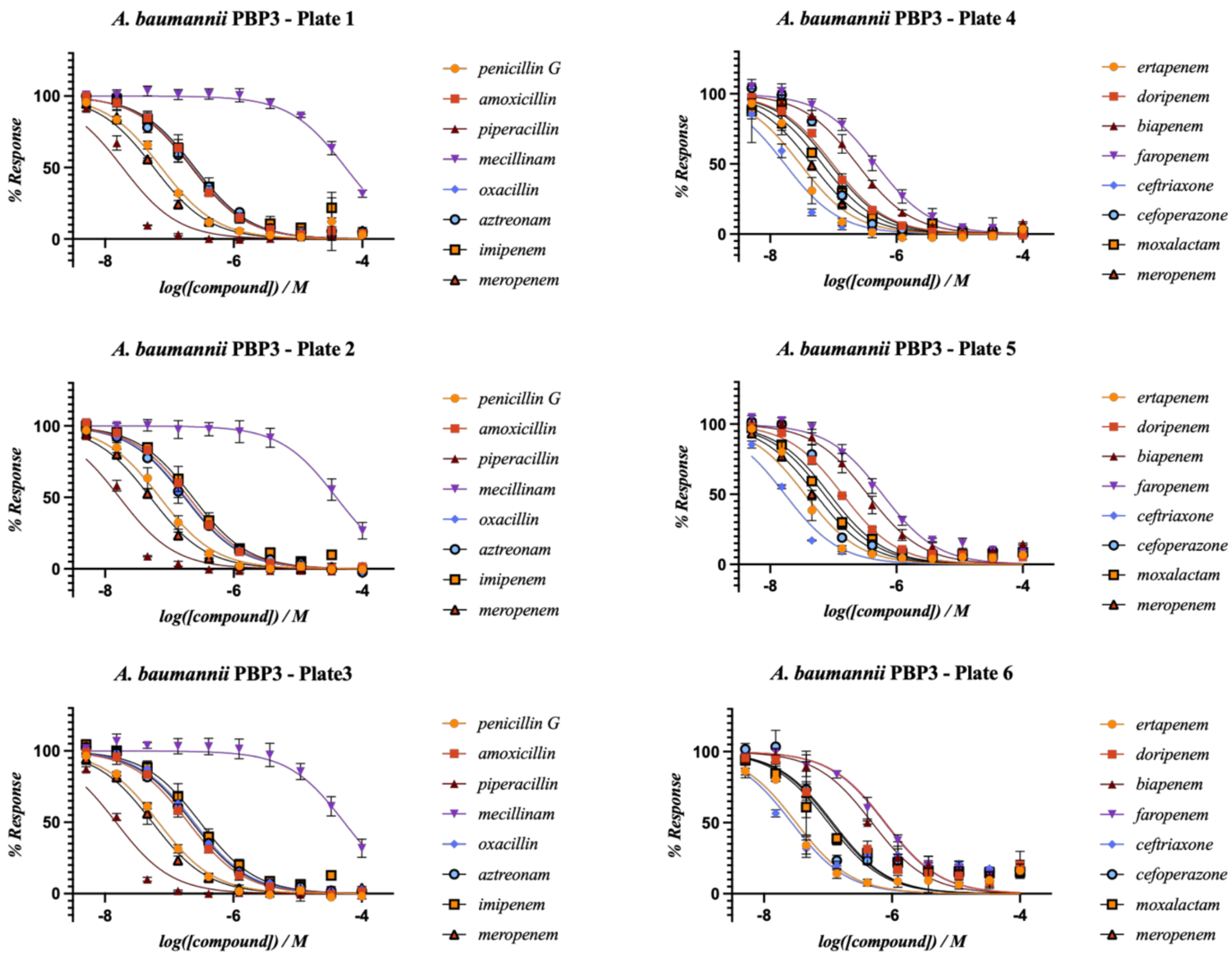

Supplementary Figure S6: Determination of the second order rate constant (k_{inact}/K_I) for inhibition of *A. baumannii* PBP3 with different covalently reacting β-lactams. Error bars represent standard deviations from three technical replicates measured simultaneously. See Figure S10 for compound structures. See Materials and Methods for assay details. The calculated value for doripenem from *A. baumannii* PBP3 plate 6 was omitted from calculations as a likely anomalous result.

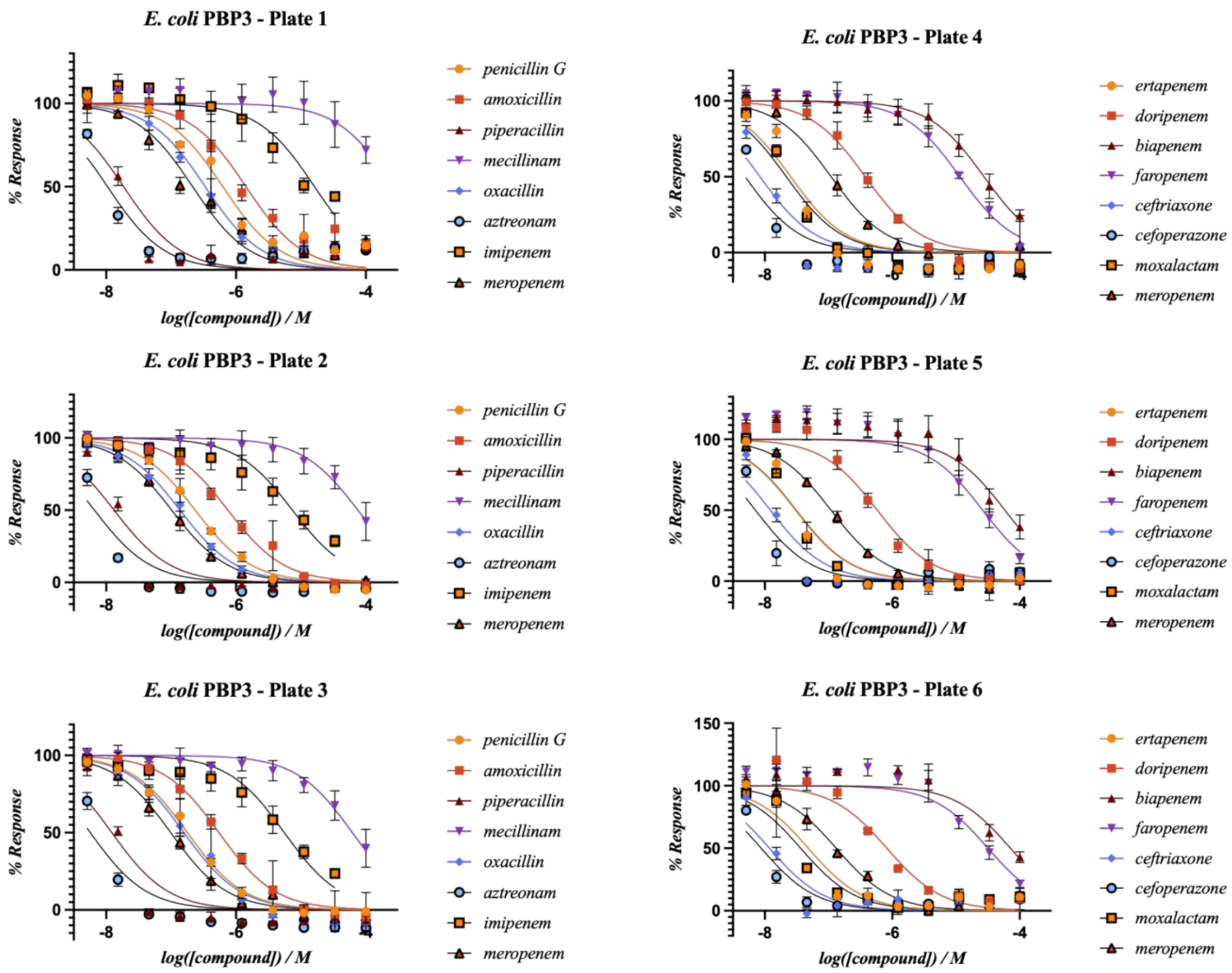

Supplementary Figure S7: Determination of the second order rate constant (k_{inact}/K_I) for inhibition of *E. coli* PBP3 with different covalently reacting β-lactams. Error bars represent standard deviations from three technical replicates measured simultaneously. See Figure S10 for compound structures. See Materials and Methods for assay details.

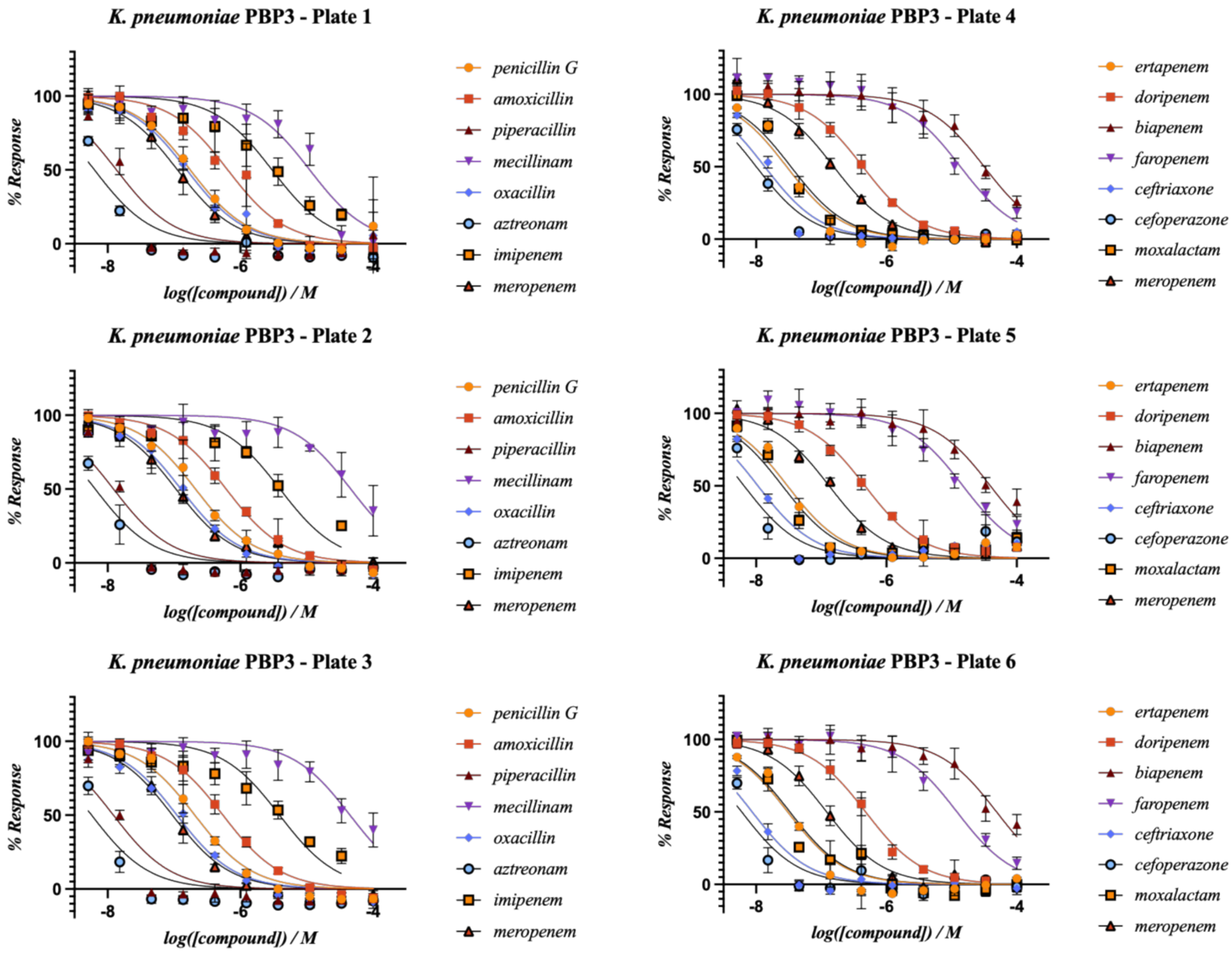

Supplementary Figure S8: Determination of the second order rate constant (k_{inact}/K_I) for inhibition of *K. pneumoniae* PBP3 with different covalently reacting β-lactams. Error bars represent standard deviations from three technical replicates measured simultaneously. See Figure S10 for compound structures. See Materials and Methods for assay details.

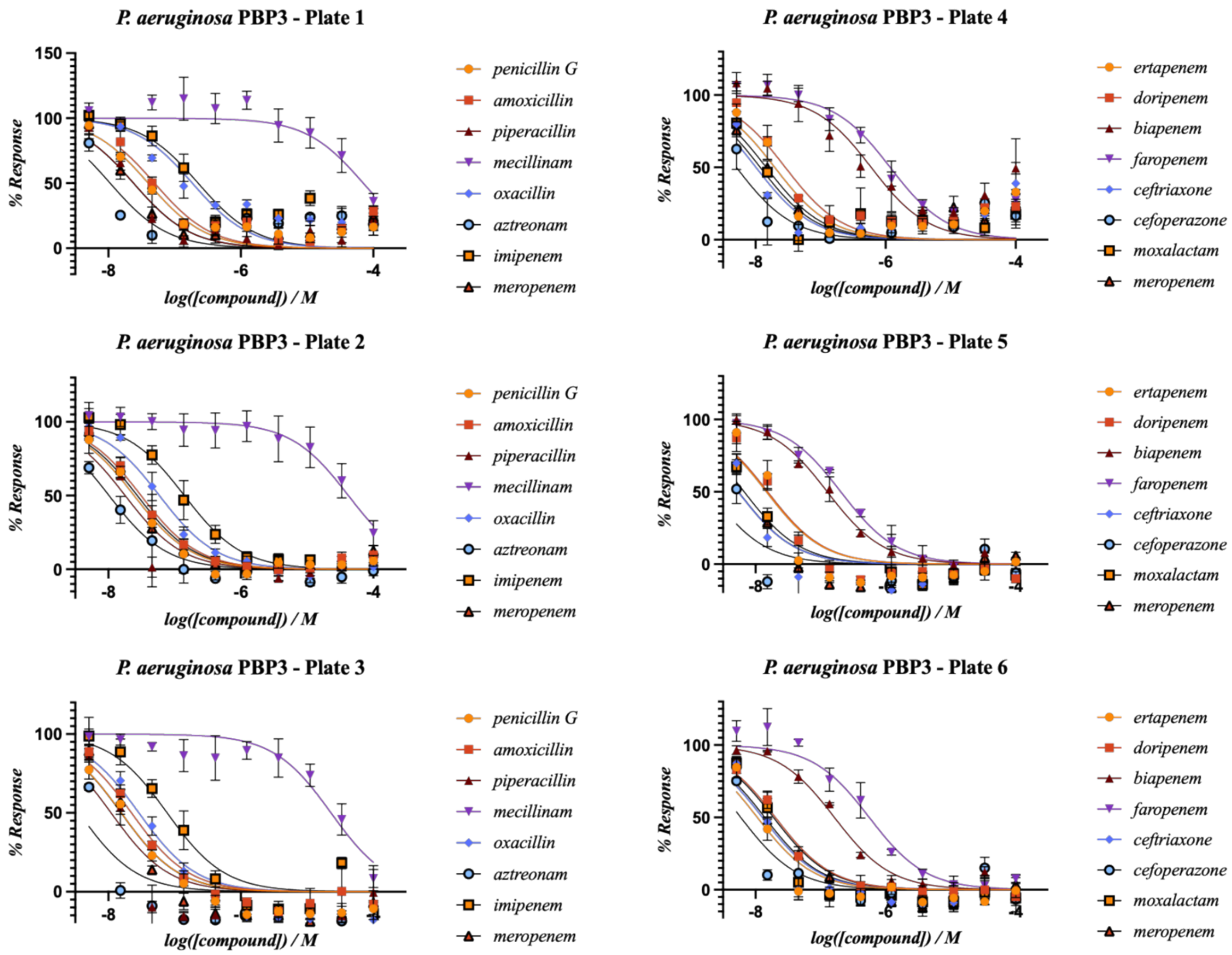

Supplementary Figure S9: Determination of the second order rate constant (k_{inact}/K_I) for inhibition of *P. aeruginosa* PBP3 with different covalently reacting β-lactams. Error bars represent standard deviations from three technical replicates measured simultaneously. See Figure S10 for compound structures. See Materials and Methods for assay details.

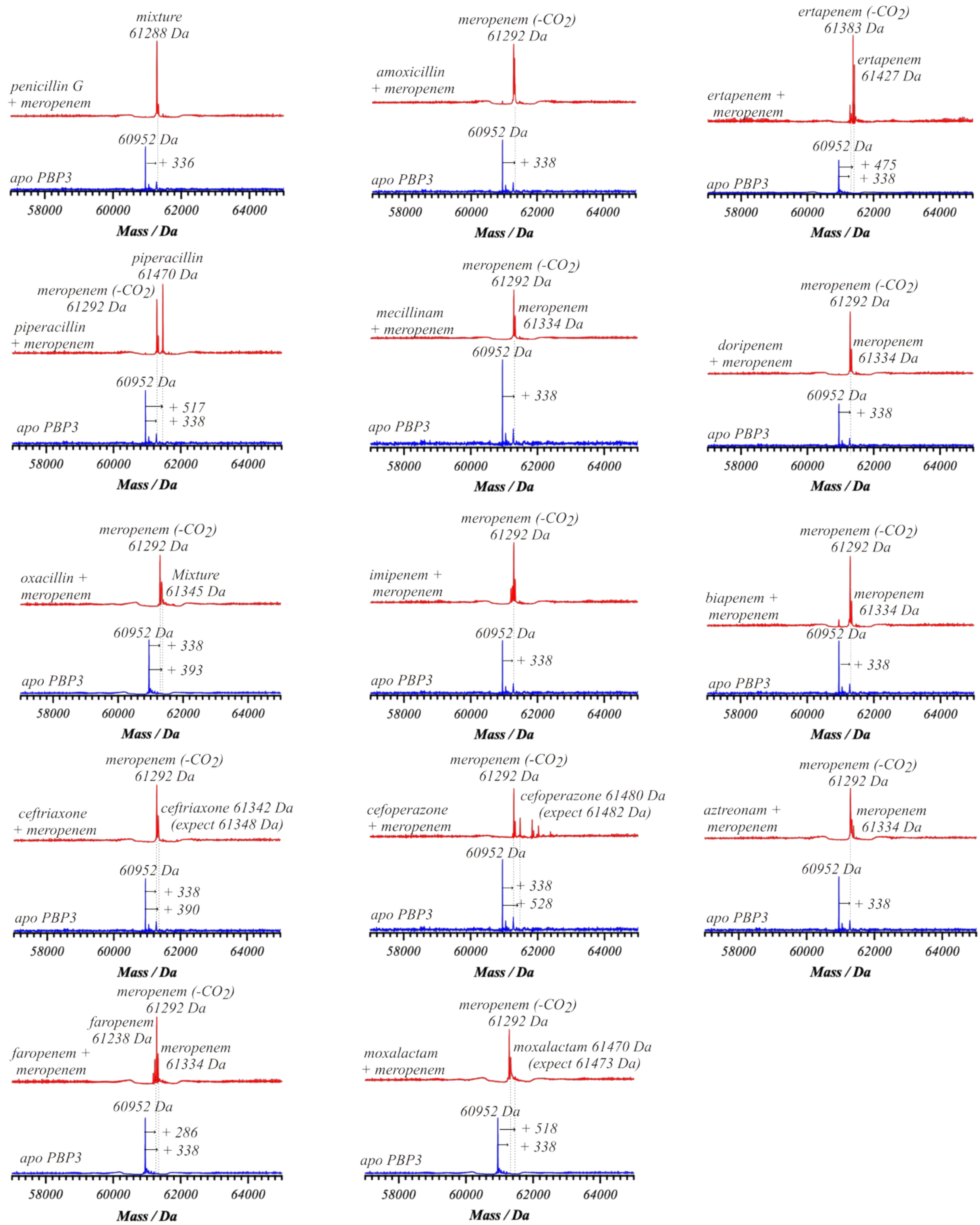

Supplementary Figure S10: Protein observed inhibition competition mass spectrometry experiments inform on the efficiency of reactions of different β-lactams with *A. baumannii* PBP3.1:1 mixtures of the inhibitors of interest and meropenem (1 mM) were added to the protein (100 μM); following incubation (10 mins) the reaction was quenched (1 % $_{v/v}$ formic acid). Each experiment was performed in duplicate. See Figure S10 for compound structures. See Materials and Methods for assay details.

Supplementary Table S1: Inhibition competition binding experiments with *A. baumannii* PBP3 analysed by UPLC-ESI-Q-TOF MS inform on the acylation efficiency in comparison to that of meropenem. 1:1 mixtures of the different inhibitors and meropenem were incubated with *A. baumannii* PBP3 (10 mins). The reaction was subsequently quenched (1 $\%_{v/v}$ formic acid) and analysed. Ceftriaxone and cefoperazone bind with loss of the cephalosporin C3' sidechain, see Supplementary Figure S12 for the proposed complexes formed upon reactions with PBP3.

Compound	Mass calculated	Mass observed	Product observed	Secondary Mass	Secondary product
meropenem	61334	61292	meropenem (- CO_2)	61334	meropenem
penicillin G	61286.11	61288	Mixture		
amoxicillin	61317.11	61292	meropenem (- CO_2)		
piperacillin	61469.17	61470	piperacillin		
mecillinam	61277.15	61292	meropenem (- CO_2)		
oxacillin	61353.11	61292	meropenem (- CO_2)		
aztreonam	61387.43	61292	meropenem (- CO_2)		
imipenem	61251.09	61292	meropenem (- CO_2)		
ertapenem	61427.14	61383	ertapenem (- CO_2)		
doripenem	61372.11	61292	meropenem (- CO_2)		
biapenem	61302.1	61292	meropenem (- CO_2)		
faropenem	61237.07	61292	meropenem (- CO_2)	61238	faropenem
ceftriaxone	61506.05	61292	meropenem (- CO_2)	61342	ceftriaxone (- C3')
cefoperazone	61597.67	61292	meropenem (- CO_2)	61480	cefoperazone (- C3')
moxalactam	61472.1	61292	meropenem (- CO_2)	61470	moxalactam

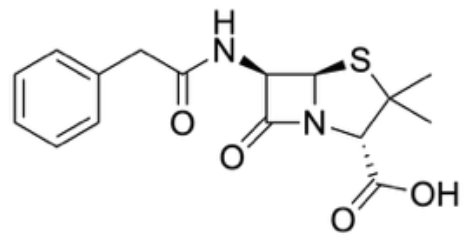

penicillin G

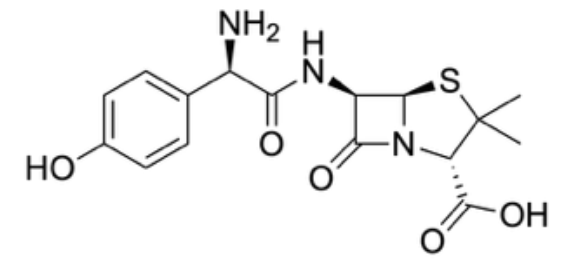

amoxicillin

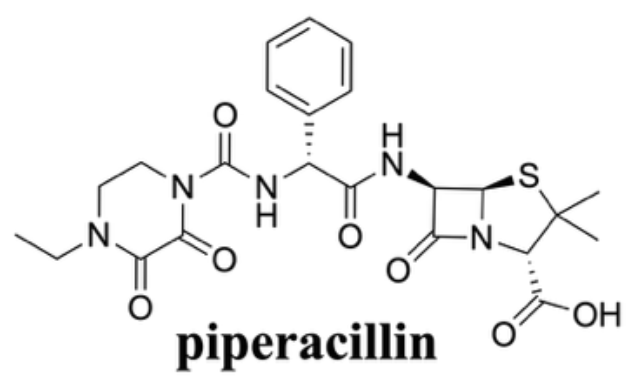

piperacillin

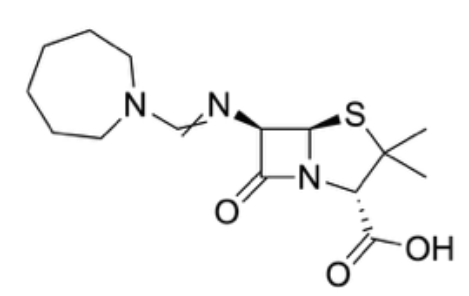

mecillinam

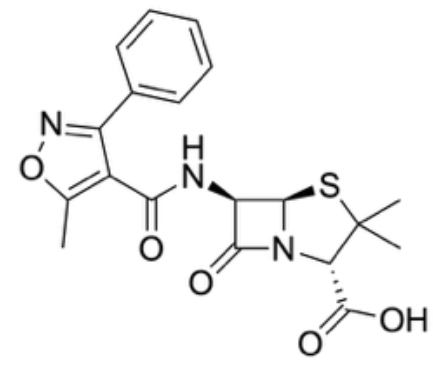

oxacillin

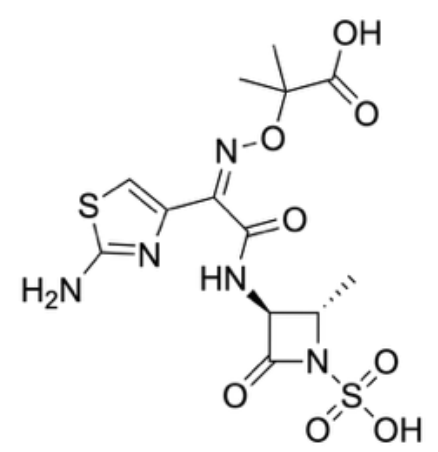

aztreonam

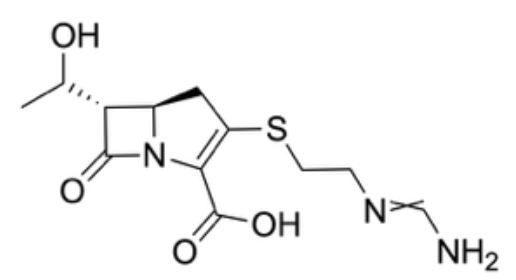

imipenem

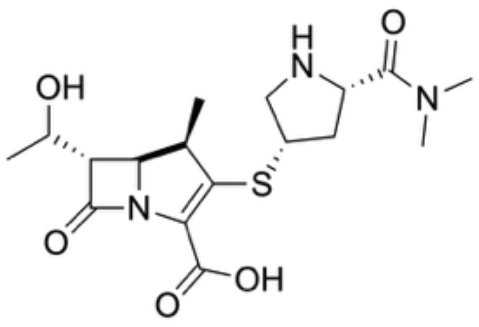

meropenem

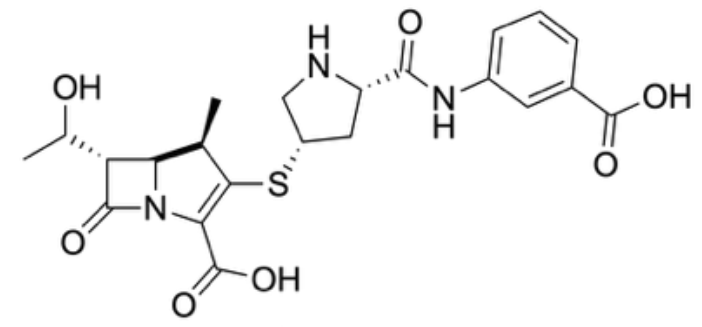

ertapenem

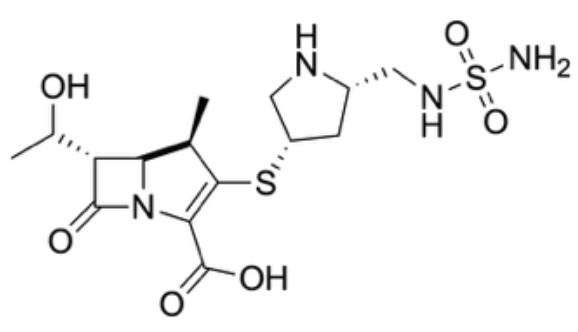

doripenem

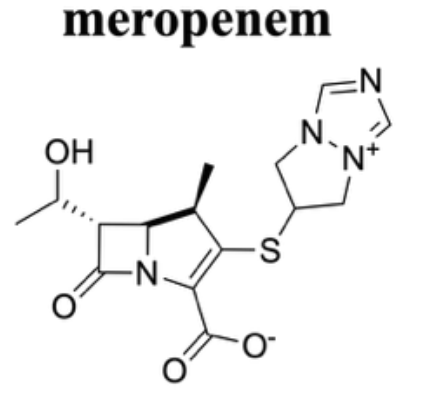

biapenem

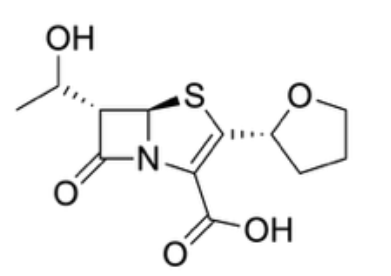

faropenem

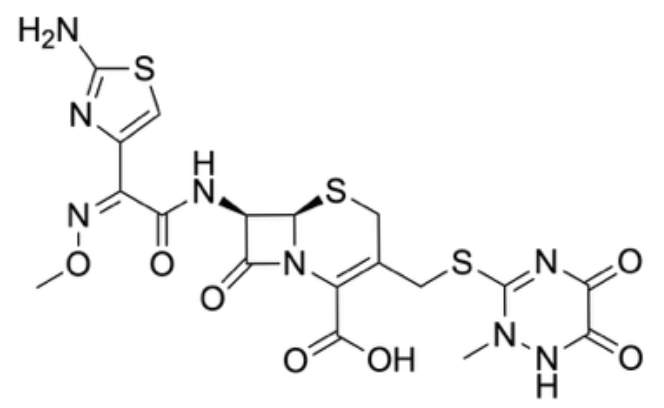

ceftriaxone

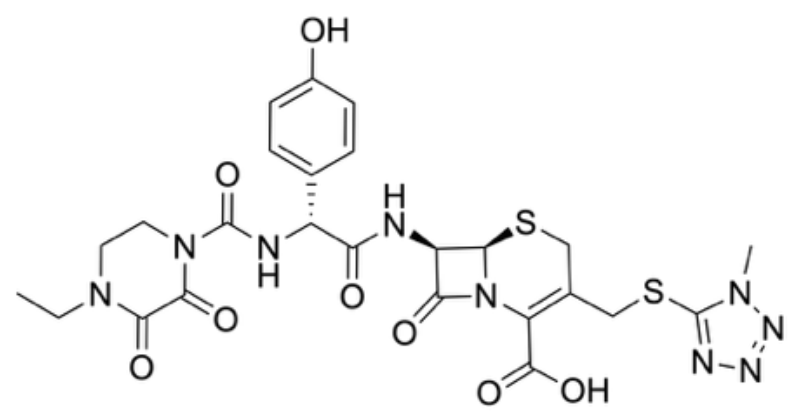

cefoperazone

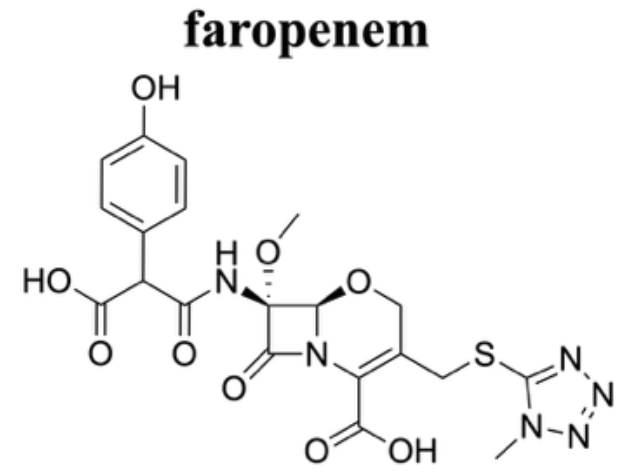

moxalactam

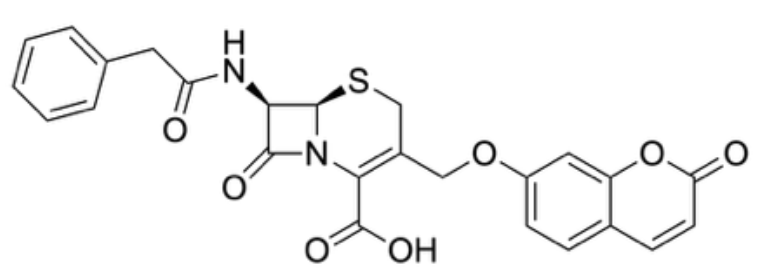

FC3

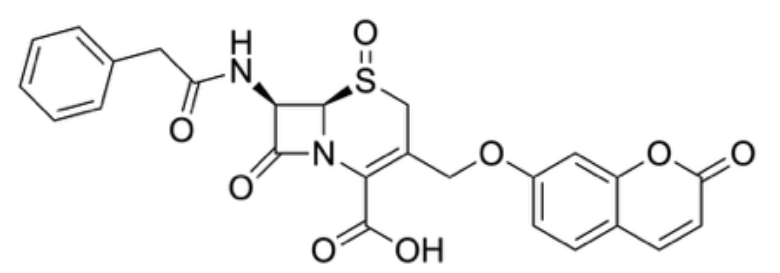

FC4

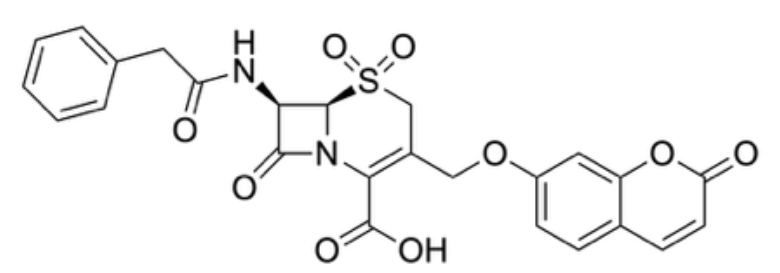

FC5

Supplementary Figure S11: Structures of β-lactam antibiotics and reporter probe compounds used in our research.

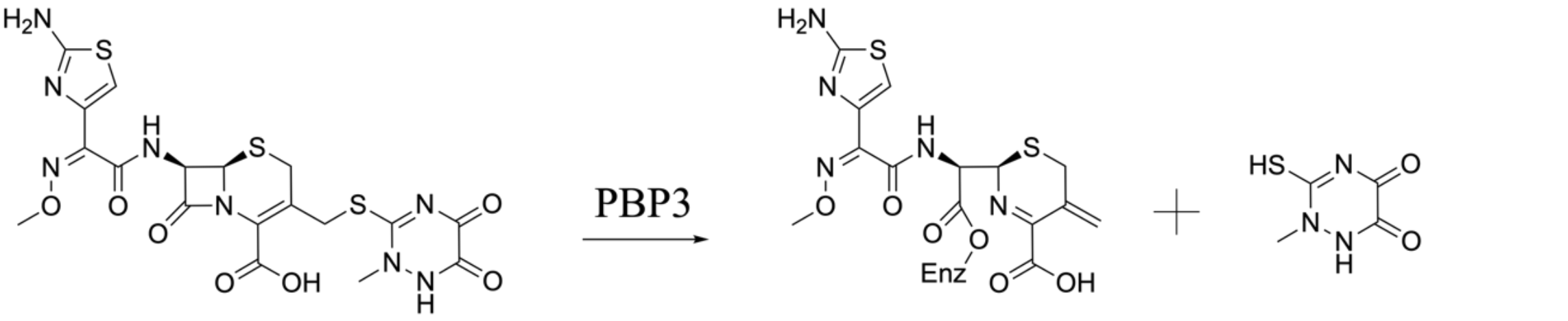

ceftriaxone
MW = 554.05 g/mol

MW = 159.01 g/mol

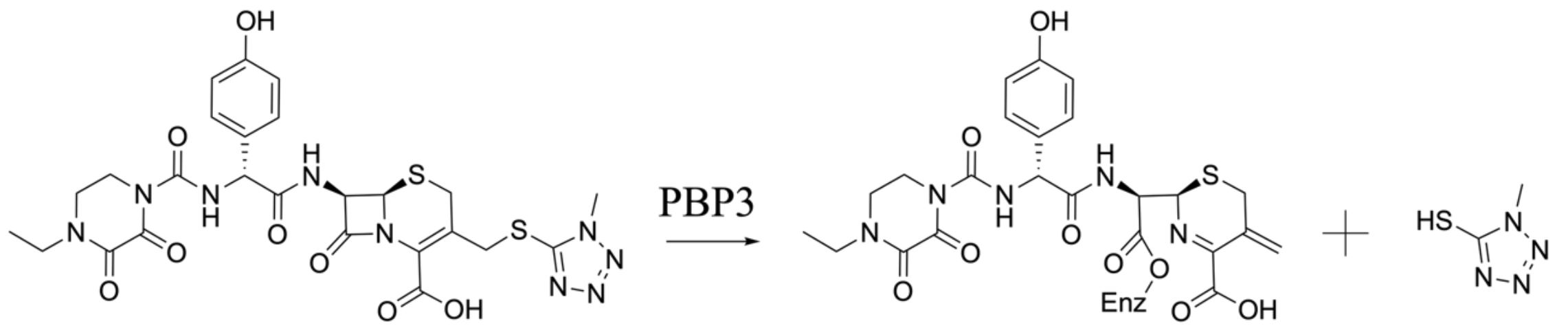

cefoperazone
MW = 645.14 g/mol

MW = 116.02 g/mol

Supplementary Figure 12: Reactions of ceftriaxone and cefoperazone with PBP3 proceed with loss of the C3' sidechain, as reflected in mass spectrometry experiments (Supplementary Figure S10 and Table S1).

Supplementary Table S2: Crystallography data collection and refinement statistics for *P. aeruginosa* PBP3 following reaction with different carbapenems.[15] Structures are deposited with the following PDB codes: 33GK (biapenem), 33GL (doripenem), 33GO (ertapenem).

	Biapenem	Doripenem	Ertapenem
**Wavelength**	0.97626	0.97626	0.97626
**Resolution range**	61.17 - 1.9 (1.95 - 1.9)	60.83 - 1.9 (1.95 - 1.9)	44.81 - 1.6 (1.64 - 1.6)
**Space group**	P 21 21 21	P 21 21 21	P 21 21 21
**Unit cell**	68.679 83.795 89.497 90 90 90	68.507 83.013 89.404 90 90 90	68.717 83.196 89.612 90 90 90
**Total reflections**	556729 (39023)	554334 (40226)	917576 (64517)
**Unique reflections**	41265 (2823)	40814 (2858)	68384 (4780)
**Multiplicity**	13.5 (13.8)	13.6 (14.1)	13.4 (13.5)
**Completeness (%)**	99.57 (97.27)	99.74 (98.65)	99.86 (99.27)
**Mean I/sigma(I)**	7.11 (0.76)	9.65 (0.82)	14.72 (0.73)
**Wilson B-factor**	31.21	32.25	26.21
**R-merge**	0.1544 (0.8458)	0.1213 (1.588)	0.07345 (1.555)
**R-meas**	0.1605 (0.878)	0.1261 (1.647)	0.07636 (1.615)
**R-pim**	0.0435 (0.2339)	0.03417 (0.4346)	0.02072 (0.4365)
**CC1/2**	0.995 (0.743)	0.999 (0.777)	1 (0.749)
**CC***	0.999 (0.923)	1 (0.935)	1 (0.925)
**Reflections used in refinement**	41180 (2811)	40747 (2846)	68305 (4764)
**Reflections used for R-free**	1941 (110)	1955 (126)	1975 (161)
**R-work**	0.1888 (0.3743)	0.1975 (0.3361)	0.1905 (0.3756)
**R-free**	0.2290 (0.3912)	0.2422 (0.3917)	0.2237 (0.3931)
**Number of non-hydrogen atoms**	4285	4139	4167
**macromolecules**	3869	3877	3897
**ligands**	24	27	39
**solvent**	392	235	231
**Protein residues**	504	507	504
**RMS(bonds)**	0.008	0.007	0.007
**RMS(angles)**	0.9	0.97	0.9
**Ramachandran favored (%)**	97.2	97.22	97.4
**Ramachandran allowed (%)**	2.8	2.78	2.6
**Ramachandran outliers (%)**	0	0	0
**Rotamer outliers (%)**	0.49	0.74	0.73
**Clashscore**	3.95	3.95	3.03
**Average B-factor**	39.19	45.53	33.78
**macromolecules**	38.96	45.71	33.31
**ligands**	63.18	45.42	50.17
**solvent**	39.95	42.55	38.91

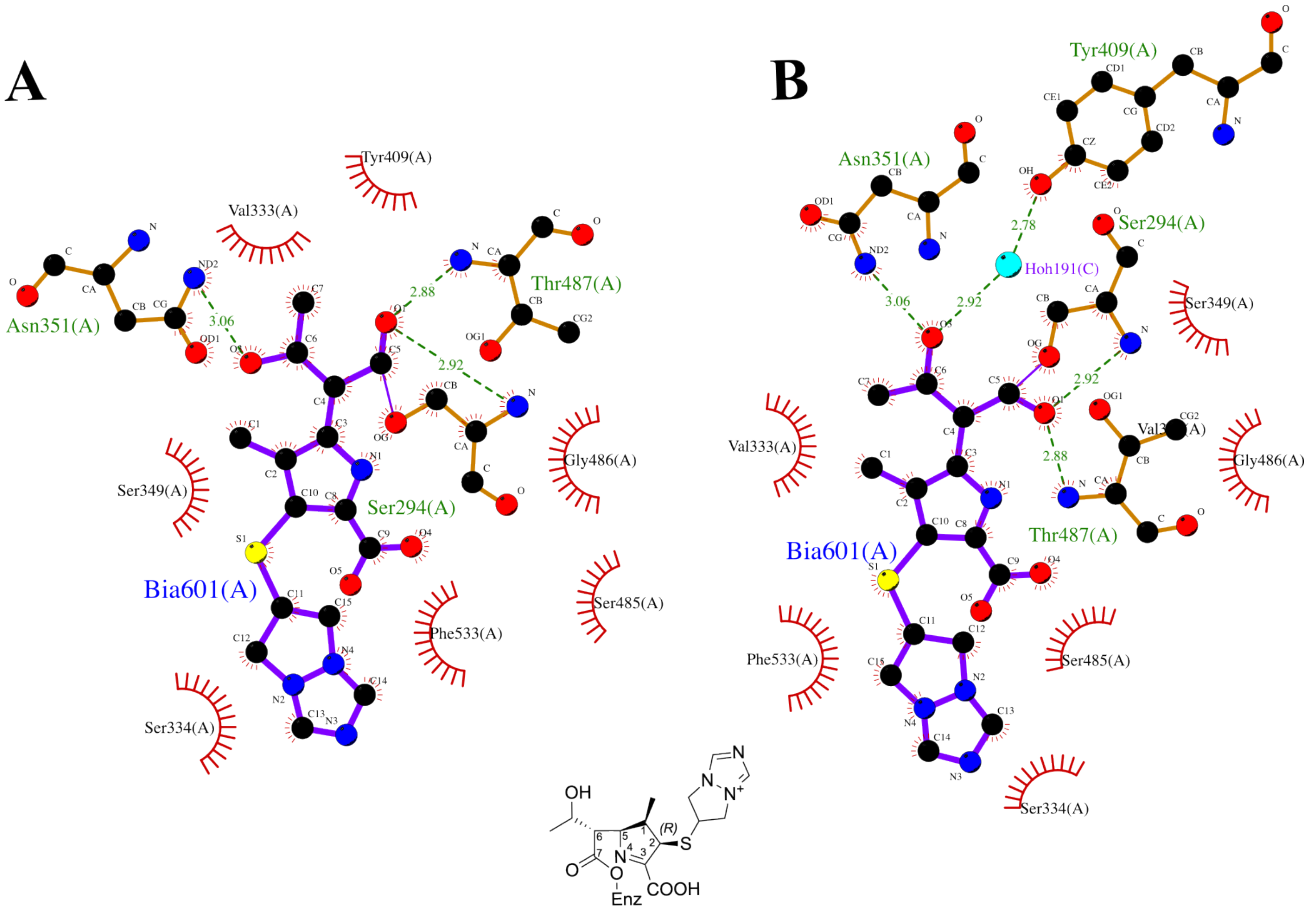

biapenem

Supplementary Figure 13: LigPlot+ schematics displaying contacts formed between: **A.** biapenem and residues within the *P. aeruginosa* PBP3 active site; **B.** biapenem, water and residues within the *P. aeruginosa* PBP3 active site. The figure was generated using LigPlot+ version 2.2.[16] See Supplementary Table S3 for the number and type of interactions formed by each ligand in the acyl enzyme complexes of *P. aeruginosa* PBP3 with different carbapenems. The structure is deposited with PDB code 33GK.

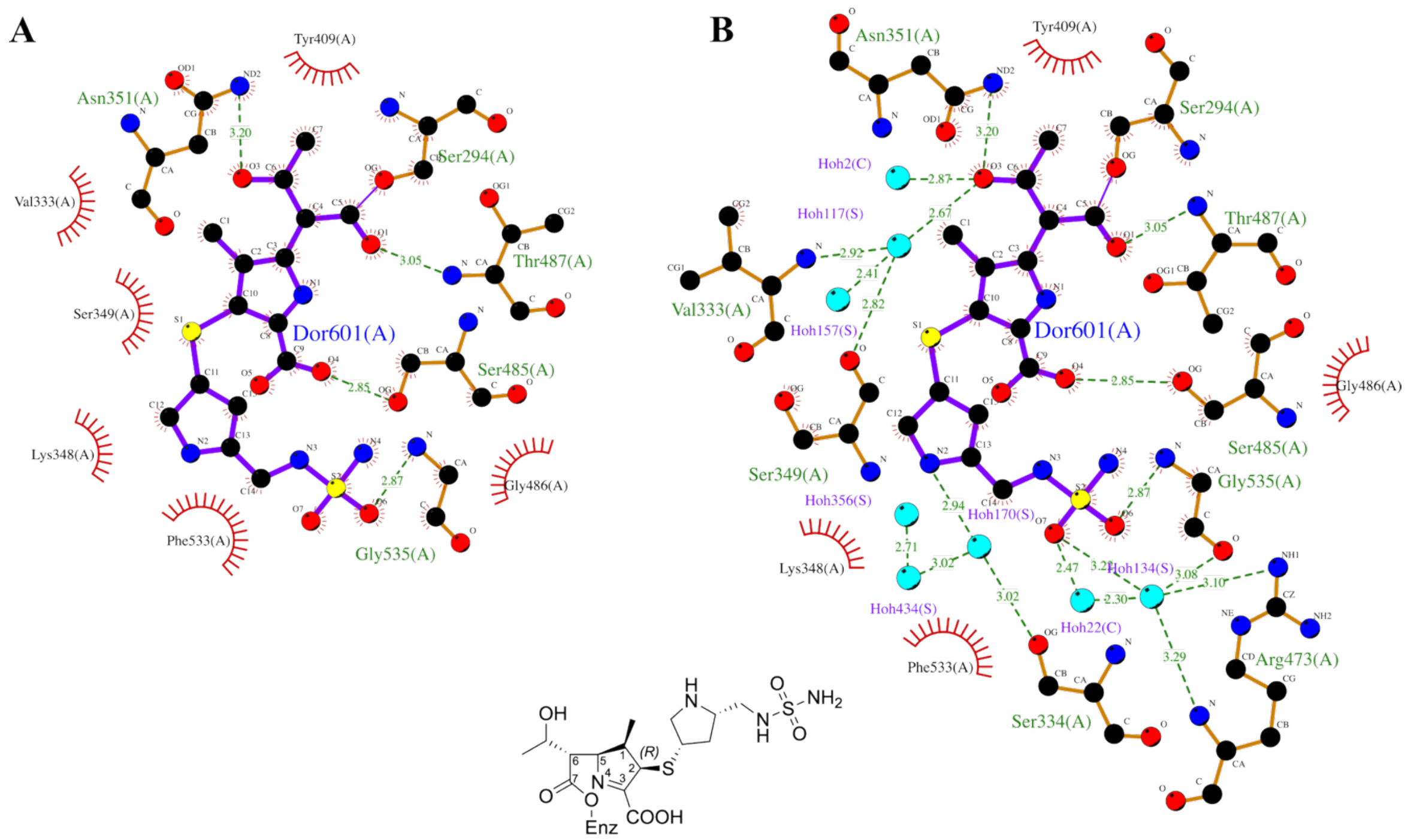

doripenem

Supplementary Figure 14: LigPlot+ schematics displaying contacts formed between: **A.** doripenem and residues within the *P. aeruginosa* PBP3 active site; **B.** doripenem, water and residues within the *P. aeruginosa* PBP3 active site. The figure was generated using LigPlot+ version 2.2.[16] See Supplementary Table S3 for the number and type of interactions formed by each ligand in the acyl enzyme complexes of *P. aeruginosa* PBP3 with different carbapenems. The structure is deposited with the PDB code 33GL.

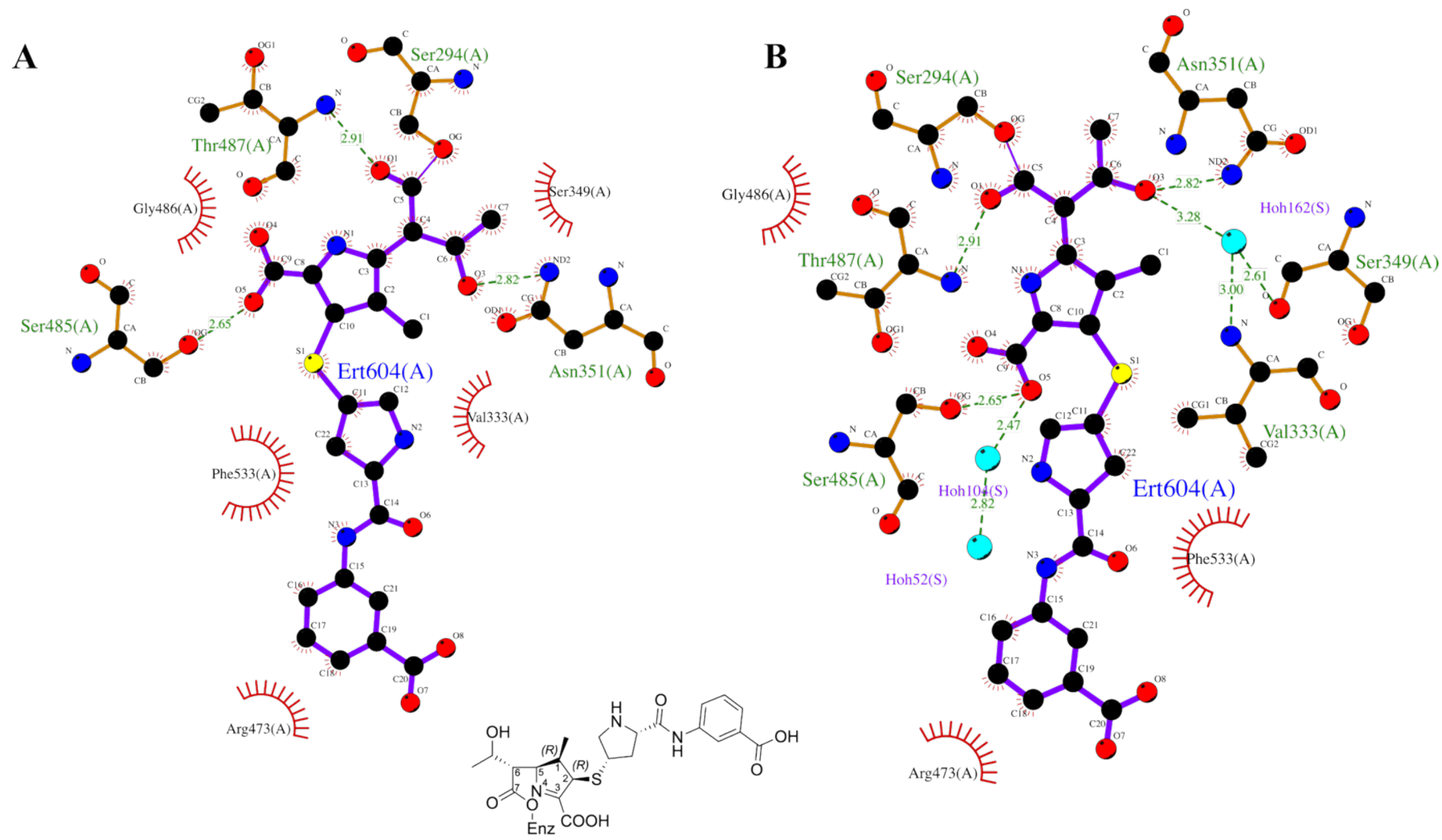

ertapenem

Supplementary Figure 15: LigPlot+ schematics displaying contacts formed between: **A.** ertapenem and residues within the *P. aeruginosa* PBP3 active site; **B.** ertapenem, water and residues within the *P. aeruginosa* PBP3 active site. The figure was generated using LigPlot+ version 2.2.[16–18] See Supplementary Table S3 for the number and type of interactions formed by each ligand in the acyl enzyme complexes of *P. aeruginosa* PBP3 with different carbapenems. The structure is deposited with the PDB code 33GO.

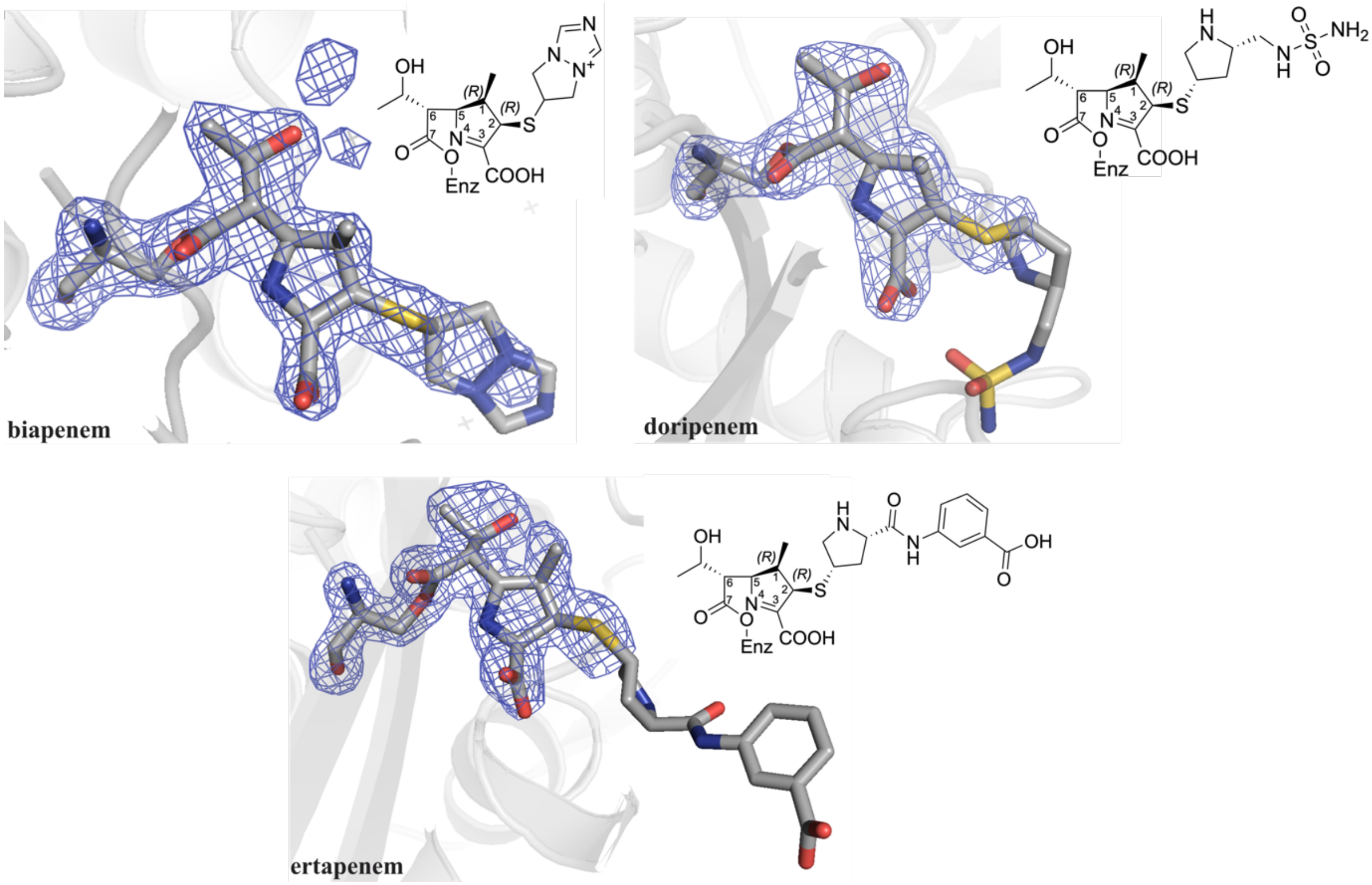

Supplementary Figure 16: Polder OMIT maps[19] contoured at 5σ showing electron density for *P. aeruginosa* PBP3 acyl-enzyme complexes derived from reactions with biapenem, doripenem, and ertapenem. The acyl-enzyme complexes were refined in their imine forms with an sp^2 C2 carbon ((2*R*)-stereochemistry), as observed for other PBP3-carbapenem derived complexes. [17,18] Structures are deposited with the following PDB codes: 33GK, 33GL, 33GO. Although the AECs were modelled with the imine form, it cannot be ruled out the enamine or (2*S*)-imine tautomeric forms are relevant in solution, an issue studied in more detail with serine-β-lactamases.[17,18] See Supplementary Table S3 for the number and type of interactions formed by each ligand in the acyl enzyme complexes of *P. aeruginosa* PBP3 with different carbapenems.

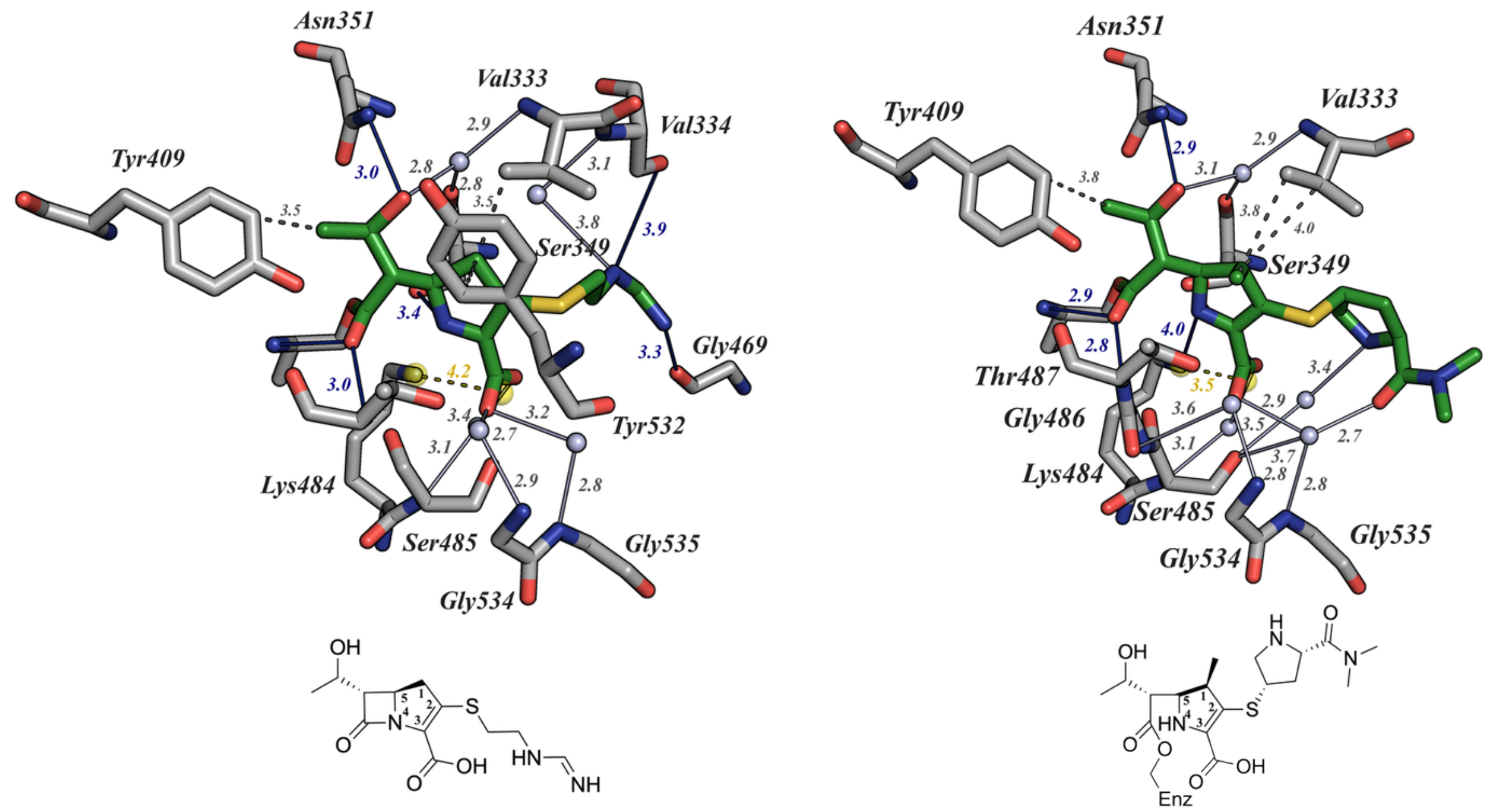

imipenem meropenem

Supplementary Figure 17: Reported crystallographic studies reveal key interactions in the acyl-enzyme complexes derived from the carbapenems imipenem (left, PDB: 3PBQ) and meropenem (right, PDB: 3PBR) with *P. aeruginosa* PBP3. Note the different interactions with the active site as a consequence of the different C2 groups.[20] Although the AECs were modelled in their enamine form it cannot be ruled out the one or both of the possible imine tautomeric forms are present at low levels and/or relevant in solution, an issue that has been studied in more detail with serine β-lactamases. [17,18] See Supplementary Table S3 for the number and type of interactions formed by each ligand in the acyl enzyme complexes of *P. aeruginosa* PBP3 with different carbapenems.

Supplementary Table S3: Number and type of interactions formed by different inhibitors in the acyl enzyme complexes of *P. aeruginosa* PBP3 with different carbapenems.[20] Interactions were identified using Protein Ligand Interaction Profiler (PLIP).[21]

Compound	biapenem	doripenem	ertapenem	meropenem	imipenem
PDB	33GK	33GL	33GO	3PBR	3PBO
Hydrogen bonds	5	9	8	5	7
Water bridges	6	4	4	9	7
Salt bridges	1	1	2	1	1
Hydrophobic interactions	3	2	2	3	3
Total	15	16	16	18	18

Supplementary References

1 S. S. van Berkel, J. Brem, A. M. Rydzik, R. Salimraj, R. Cain, A. Verma, R. J. Owens, C. W. G. Fishwick, J. Spencer and C. J. Schofield, *J. Med. Chem.*, 2013, **56**, 6945–6953.

2 H. G. Smith, S. Basak, V. Aniebok, M. J. Beech, F. M. Alshref, M. D. Allen, A. J. M. Farley and C. J. Schofield, *Chem. Sci.*, 2024, **15**, 16928–16937.

3 S. Han, N. Caspers, R. P. Zaniewski, B. M. Lacey, A. P. Tomaras, X. Feng, K. F. Geoghegan and V. Shanmugasundaram, *J. Am. Chem. Soc.*, 2011, **133**, 20536–20545.

4 M. de Munnik, P. A. Lang, F. De Dios Anton, M. Cacho, R. H. Bates, J. Brem, B. Rodríguez Miquel and C. J. Schofield, *Chem. Sci.*, 2023, **14**, 7262–7278.

5 I. Miyahisa, T. Sameshima and M. S. Hixon, *Angewandte Chemie*, 2015, **127**, 14305–14308.

6 G. Winter, D. G. Waterman, J. M. Parkhurst, A. S. Brewster, R. J. Gildea, M. Gerstel, L. Fuentes-Montero, M. Vollmar, T. Michels-Clark, I. D. Young, N. K. Sauter and G. Evans, *Acta Crystallogr. D Struct. Biol.*, 2018, **74**, 85–97.

7 A. J. McCoy, R. W. Grosse-Kunstleve, P. D. Adams, M. D. Winn, L. C. Storoni and R. J. Read, *J. Appl. Crystallogr.*, 2007, **40**, 658–674.

8 J. Jumper, R. Evans, A. Pritzel, T. Green, M. Figurnov, O. Ronneberger, K. Tunyasuvunakool, R. Bates, A. Žídek, A. Potapenko, A. Bridgland, C. Meyer, S. A. A. Kohl, A. J. Ballard, A. Cowie, B. Romera-Paredes, S. Nikolov, R. Jain, J. Adler, T. Back, S. Petersen, D. Reiman, E. Clancy, M. Zielinski, M. Steinegger, M. Pacholska, T. Berghammer, S. Bodenstein, D. Silver, O. Vinyals, A. W. Senior, K. Kavukcuoglu, P. Kohli and D. Hassabis, *Nature*, 2021, **596**, 583–589.

9 D. Liebschner, P. V. Afonine, M. L. Baker, G. Bunkóczi, V. B. Chen, T. I. Croll, B. Hintze, L.-W. Hung, S. Jain, A. J. McCoy, N. W. Moriarty, R. D. Oeffner, B. K. Poon, M. G. Prisant, R. J. Read, J. S. Richardson, D. C. Richardson, M. D. Sammito, O. V. Sobolev, D. H. Stockwell, T. C. Terwilliger, A. G. Urzhumtsev, L. L. Videau, C. J. Williams and P. D. Adams, *Acta Crystallogr. D Struct. Biol.*, 2019, **75**, 861–877.

10 P. Emsley, B. Lohkamp, W. G. Scott and K. Cowtan, *Acta Crystallogr. D Biol. Crystallogr.*, 2010, **66**, 486–501.

11 F. Long, R. A. Nicholls, P. Emsley, S. Gražulis, A. Merkys, A. Vaitkus and G. N. Murshudov, *Acta Crystallogr. D Struct. Biol.*, 2017, **73**, 103–111.

12 F. Long, R. A. Nicholls, P. Emsley, S. Gražulis, A. Merkys, A. Vaitkus and G. N. Murshudov, *Acta Crystallogr. D Struct. Biol.*, 2017, **73**, 112–122.

13 J. Agirre, M. Atanasova, H. Bagdonas, C. B. Ballard, A. Baslé, J. Beilsten-Edmands, R. J. Borges, D. G. Brown, J. J. Burgos-Mármol, J. M. Berrisford, P. S. Bond, I. Caballero, L. Catapano, G. Chojnowski, A. G. Cook, K. D. Cowtan, T. I. Croll, J. É. Debreczeni, N. E. Devenish, E. J. Dodson, T. R. Drevon, P. Emsley, G. Evans, P. R. Evans, M. Fando, J. Foadi, L. Fuentes-Montero, E. F. Garman, M. Gerstel, R. J. Gildea, K. Hatti, M. L. Hekkelman, P. Heuser, S. W. Hoh, M. A. Hough, H. T. Jenkins, E. Jiménez, R. P. Joosten, R. M. Keegan, N. Keep, E. B. Krissinel, P. Kolenko, O. Kovalevskiy, V. S. Lamzin, D. M. Lawson, A. A. Lebedev, A. G. W. Leslie, B. Lohkamp, F. Long, M. Malý, A. J. McCoy, S. J. McNicholas, A. Medina, C. Millán, J. W. Murray, G. N. Murshudov, R. A. Nicholls, M. E. M. Noble, R. Oeffner, N. S. Pannu, J. M. Parkhurst, N. Pearce, J. Pereira, A. Perrakis, H. R. Powell, R. J. Read, D. J. Rigden, W. Rochira, M. Sammito, F. Sánchez Rodríguez, G. M. Sheldrick, K. L. Shelley, F. Simkovic, A. J. Simpkin, P. Skubak, E. Sobolev, R. A. Steiner, K. Stevenson, I. Tews, J. M. H. Thomas, A. Thorn, J. T. Valls, V. Uski, I. Usón, A. Vagin, S. Velankar, M. Vollmar, H. Walden, D. Waterman, K. S. Wilson, M. D. Winn, G.

Winter, M. Wojdyr and K. Yamashita, *Acta Crystallogr. D Struct. Biol.*, 2023, **79**, 449–461.

14 K. F. Geoghegan, H. B. F. Dixon, P. J. Rosner, L. R. Hoth, A. J. Lanzetti, K. A. Borzilleri, E. S. Marr, L. H. Pezzullo, L. B. Martin, P. K. LeMotte, A. S. McColl, A. V. Kamath and J. G. Stroh, *Anal. Biochem.*, 1999, **267**, 169–184.

15 D. Liebschner, P. V. Afonine, M. L. Baker, G. Bunkoczi, V. B. Chen, T. I. Croll, B. Hintze, L. W. Hung, S. Jain, A. J. McCoy, N. W. Moriarty, R. D. Oeffner, B. K. Poon, M. G. Prisant, R. J. Read, J. S. Richardson, D. C. Richardson, M. D. Sammito, O. V. Sobolev, D. H. Stockwell, T. C. Terwilliger, A. G. Urzhumtsev, L. L. Videau, C. J. Williams and P. D. Adams, *Acta Crystallogr. D Struct. Biol.*, 2019, **75**, 861–877.

16 R. A. Laskowski and M. B. Swindells, *J. Chem. Inf. Model.*, 2011, **51**, 2778–2786.

17 C. T. Lohans, E. I. Freeman, E. van Groesen, C. L. Tooke, P. Hinchliffe, J. Spencer, J. Brem and C. J. Schofield, *Sci. Rep.*, 2019, **9**, 13608.

18 A. V. Krivitskaya and M. G. Khrenova, *J. Chem. Inf. Model.*, 2022, **62**, 6519–6529.

19 D. Liebschner, P. V. Afonine, N. W. Moriarty, B. K. Poon, O. V. Sobolev, T. C. Terwilliger and P. D. Adams, *Acta Crystallogr. D Struct. Biol.*, 2017, **73**, 148–157.

20 S. Han, R. P. Zaniewski, E. S. Marr, B. M. Lacey, A. P. Tomaras, A. Evdokimov, J. R. Miller and V. Shanmugasundaram, *Proceedings of the National Academy of Sciences*, 2010, **107**, 22002–22007.

21 P. Schake, S. N. Bolz, K. Linnemann and M. Schroeder, *Nucleic Acids Res.*, 2025, **53**, W463–W465.
